# Cellular and axonal transport phenotypes due to the *C9ORF72* HRE in iPSC motor and sensory neurons

**DOI:** 10.1016/j.stemcr.2024.05.008

**Published:** 2024-06-13

**Authors:** Jakub Scaber, Iona Thomas-Wright, Alex J. Clark, Yinyan Xu, Björn F. Vahsen, Mireia Carcolé, Ruxandra Dafinca, Lucy Farrimond, Adrian M. Isaacs, David L. Bennett, Kevin Talbot

**Affiliations:** 1 Nuffield Department of Clinical Neurosciences, University of Oxford, John Radcliffe Hospital, OX3 9DU Oxford, UK; 2 Kavli Institute for Nanoscience Discovery, University of Oxford, Dorothy Crowfoot Hodgkin Building, OX1 3QU Oxford, UK; 3 Centre for Neuroscience, Surgery and Trauma, Blizard Institute, Queen Mary University, E1 2AT London, UK; 4 Chinese Academy of Medical Sciences (CAMS), CAMS Oxford Institute (COI), Nuffield Department of Medicine, University of Oxford, OX3 7FZ Oxford, UK; 5 UK Dementia Research Institute at UCL and Department of Neurodegenerative Disease, UCL Queen Square Institute of Neurology, WCIN 3BG London, UK

**Keywords:** amyotrophic lateral sclerosis, motor neuron, sensory neuron, selective vulnerability, induced pluripotent stem cells

## Abstract

Induced pluripotent stem cell (iPSC)-derived motor neurons (MNs) from patients with amyotrophic lateral sclerosis (ALS) and the *C9ORF72* hexanucleotide repeat expansion (HRE) have multiple cellular phenotypes, but which of these accurately reflect the biology underlying the cell-specific vulnerability of ALS is uncertain. We therefore compared phenotypes due to the *C9ORF72* HRE in MNs with sensory neurons (SNs), which are relatively spared in ALS. The iPSC models were able to partially reproduce the differential gene expression seen between adult SNs and MNs. We demonstrated that the typical hallmarks of *C9ORF72*-ALS, including RNA foci and dipeptide formation, as well as specific axonal transport defects, occurred equally in MNs and SNs, suggesting that these *in vitro* phenotypes are not sufficient to explain the cell-type selectivity of ALS in isolation.

## Highlights

• iPSC motor (MN) & sensory neurons (SN) allow studying selective vulnerability in ALS
• The iPSC model partly reproduced differential expression between adult MN & SN
• *C9ORF72* RNA foci, dipeptides & axonal transport slowing were seen in iPSC MN & SN
• These phenotypes unlikely explain the cell-type selectivity of ALS in isolation

## Teaser

Scaber et al. investigate the cell-type specificity of phenotypes seen in induced pluripotent stem cell (iPSC)-derived motor neurons with the *C9ORF72* expansion by comparing them to sensory neurons. RNA foci, dipeptides, and axonal transport slowing were equally seen in both cell types, and these phenotypes unlikely explain the cell-type selectivity of amyotrophic lateral sclerosis in isolation.

## Introduction

Amyotrophic lateral sclerosis (ALS) is a currently incurable neurodegenerative disease with a lifetime risk of 1:400 (Johnston et al., 2006). Its hallmark is the preferential degeneration of motor neurons (MNs) in the spinal cord and Betz cells in the corticospinal tract, with relative sparing of other cell types such as sensory neurons (SNs) (Kawamura et al., 1981), the visual system (Geser et al., 2008), the oculomotor nucleus (Okamoto et al., 1993), and the pelvic floor motor system (Kihira et al., 1997). The molecular basis for this anatomical selectivity remains obscure and may be due to cell autonomous effects in MNs or contributions from non-neuronal cell types to MN degeneration (Vahsen et al., 2021) or arise from the inherent vulnerability of the network architecture of the voluntary motor system (Talbot, 2014).

ALS is a clinically, pathologically, and genetically heterogeneous condition (Feldman et al., 2022), but the identification of multiple autosomal dominantly inherited variants of high penetrance has allowed the study of specific molecular subtypes. The commonest of these is a hexanucleotide expansion (HRE) in the first intron of *C9ORF72*, which accounts for up to 50% of all ALS cases with a family history of ALS or frontotemporal dementia. *C9ORF72* HRE-positive ALS causes typical TAR DNA-binding protein 43 (TDP-43) positive pathology but also results in a reduction in *C9ORF72* transcript levels, accumulation of repeat RNA in intracellular foci, and inclusions of dipeptide protein aberrantly translated from the hexanucleotide RNA. Induced pluripotent stem cell (iPSC) MNs have successfully recapitulated these *C9ORF72* HRE-dependent features with the qualification that dipeptide protein can be detected in cell lysates, but iPSC MNs do not have intracellular dipeptide inclusions (Braems et al., 2020). In addition, several disease-specific cellular phenotypes have been reported, including reduced survival, activation of autophagy markers (Braems et al., 2020), altered stress granule dynamics (Abo-Rady et al., 2020; Dafinca et al., 2016), impaired axonal transport (Abo-Rady et al., 2020; Fumagalli et al., 2021; Mehta et al., 2021), and nucleocytoplasmic transport (Zhang et al., 2015). However, key pathological hallmarks of ALS, mislocalization of TDP-43 or formation of cytoplasmic inclusions, have not been reliably demonstrated.

Thus far, modeling of ALS using iPSCs has predominantly focused on cell types thought to be selectively affected by ALS or frontotemporal dementia, such as MNs, cortical neurons, and glial cells (Giacomelli et al., 2022). A complementary approach, using iPSCs to differentiate cell types which are relatively spared in ALS, has the potential to provide insights into intrinsic vulnerability and resistance in disease. In ALS, SNs are an ideal candidate to study the specificity of iPSC MN phenotypes, as they traverse the same peripheral environment as spinal MNs and have similarly long axons. Although mild SN pathology is observed in ALS mouse models, it does not result in changes to cell numbers (Guo et al., 2009). Abnormal SN electrophysiology has been reported in human ALS patients, but the effects are largely sub-clinical in contrast to the debilitating motor symptoms caused by MN loss (Heads et al., 1991; Tao et al., 2018).

Here, we describe an in-depth phenotypic and molecular characterization of iPSC-derived SNs and MNs in parallel and investigate the effect of the *C9ORF72* HRE on both cell types, with the aim of elucidating factors that make MNs specifically vulnerable to degeneration.

## Results

### iPSC-derived MNs and SNs have divergent transcriptomes that recapitulate their cell type

**Figure 1:**
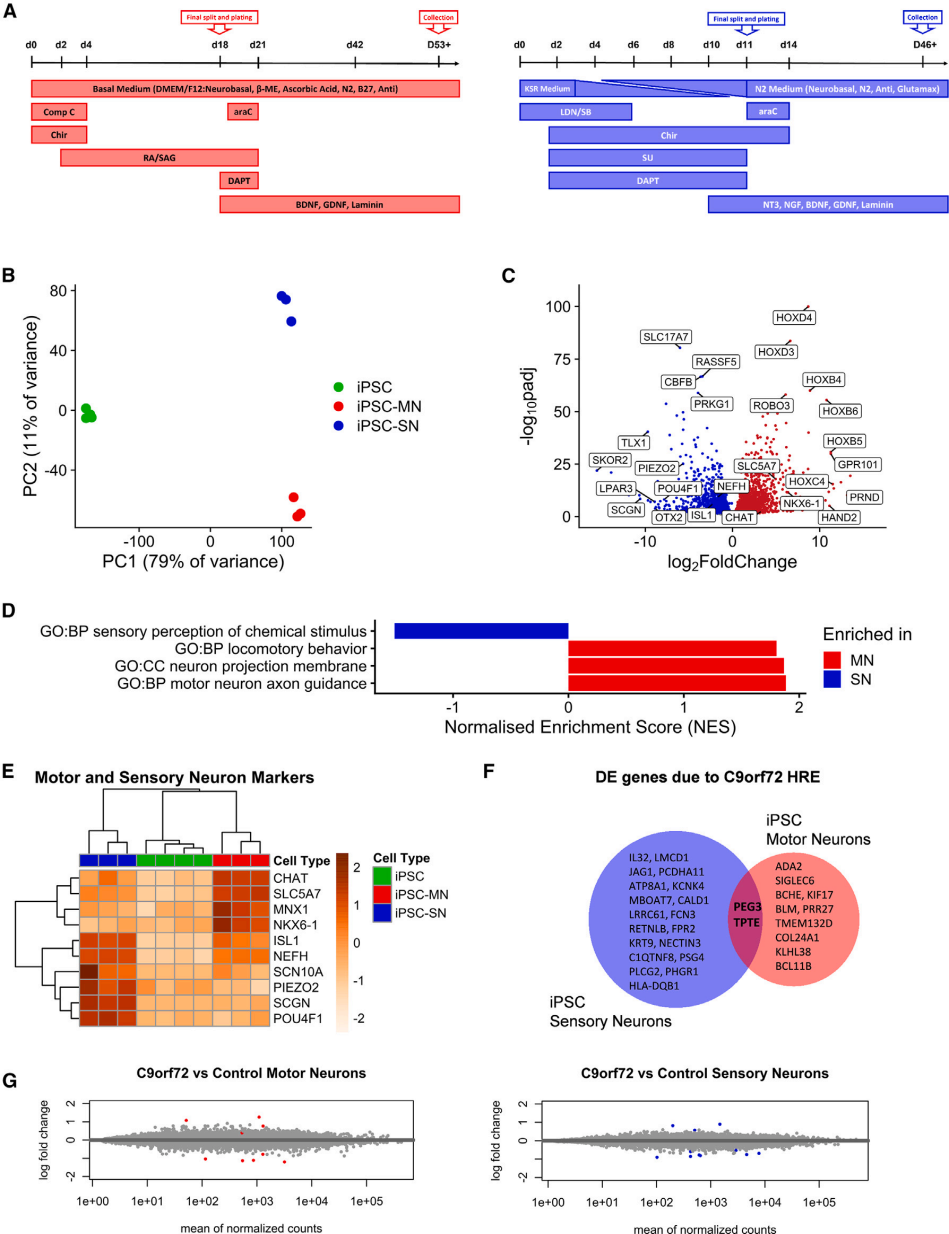
Sensory and motor neuron differentiations from iPSCs result in cell populations that recapitulate expected biological differences (A) Schematic diagrams showing sensory and motor neuron differentiations. Anti, antibiotic-antimycotic; araC, cytosine arabinoside; BDNF, brain-derived neurotrophic factor; β-ME, β-mercaptoethanol; Chir, Chir 99021; comp C, compound C; GDNF, glial cell-derived neurotrophic factors; NT3, neurotrophin 3; NGF, nerve growth factor; RA, retinoic acid; SAG, smoothened agonist. (B) Principal-component (PC) analysis of iPSC MNs, iPSC SNs, and iPSCs. (C) Volcano plot showing differentially expressed genes between iPSC MNs and iPSC SNs, with genes enriched in motor neurons in red (FDR <0.05) and those enriched in iPSC SNs in blue (FDR <0.05). (D) Significant (FDR <0.05) gene ontology enrichment in iPSC MNs and iPSC SNs showing top enriched categories after filtering for neuronally relevant terms (“senso,” “nerve,” “neuro,” “nervous,” “spinal,” “motor”). (E) Heatmap of mean normalized expression of known motor and sensory neuronal marker genes in iPSC MNs, iPSC SNs, and iPSCs. Marker genes correctly cluster into three categories: present in iPSC MNs only, present in iPSC SNs only, and present in both. (F) Venn diagram of differentially expressed genes due to the *C9ORF72* HRE in iPSC MNs and iPSC SNs. (G) MA plot of differential expression analysis between *C9ORF72*+ iPSC MNs and controls (red, FDR <0.05) and *C9ORF72*+ iPSC SNs and controls (blue, FDR <0.05).

We generated bulk RNA sequencing data from iPSC MN and iPSC SN lines derived from three healthy control lines. Cells were harvested five weeks after final plating (day *in vitro* [DIV] 53 for iPSC MN and DIV 46 for iPSC SN (Figure 1A).

The first two principal components accounted for 90% of the total variance. As expected, the first principal component separated iPSCs from iPSC MNs and iPSC SNs and accounted for 79% of total variance. The second principal component separated iPSC MNs from iPSC SNs and accounted for 11% of the variance (Figure 1B). 5,906 protein-coding genes were found to be differentially expressed between iPSC MNs and iPSC SNs (Figure 1C), and gene set enrichment analysis (GSEA) confirmed appropriate enrichment of the relevant Gene Ontology categories for both cell types, after semantically filtering for terms relevant to the nervous system (adjusted *p* < 0.05, Figure 1D).

Subsequently, we used the RNA sequencing results to visualize marker genes that are used to identify MNs and SNs. The transcription factors *NKX6.1* and *MNX1* (encoding Hb9), as well as the cholinergic markers *CHAT* (encoding choline acetyltransferase) and *SLC5A7* (encoding the choline transporter), were expressed specifically in MNs. SN-specific markers were found to be the transcription factor *POU4F1* (encoding Brn3α), the mechanosensory receptor *PIEZO2*, and the calcium-binding protein *SCGN* (encoding secretagogin). *ISL1* and *NFH* were increased in both MNs and SNs compared to iPSCs. The use of the aforementioned markers resulted in the correct supervised clustering of iPSC MNs, iPSC SNs, and iPSCs (Figure 1E).

We then compared the aforementioned control cell lines with iPSC MN and iPSC SN lines from three different *C9ORF72* HRE carriers. In contrast to the extensive differential expression changes seen between iPSC MNs and iPSC SNs, only 12 genes were differentially expressed between *C9ORF72* HRE and control iPSC MNs and 21 genes between *C9ORF72* HRE and control iPSC SNs (Figure 1F), highlighting the subtle transcriptional consequences of the pathogenic variant in this model system even after prolonged growth in culture, an observation supported by a narrow log_2_-fold change distribution in both cell types (Figure 1G). There was a significant overlap in the differential expression due to the *C9ORF72* HRE between the two cell types (2/21, *p* < 0.001), indicating a convergence of the two datasets.

### Transcriptomic comparison of iPSC neurons with human postmortem datasets reveals differences between the iPSC models and the corresponding adult cell types

iPSC-derived neurons reflect low embryonic age even after prolonged culture (Ho et al., 2021); thus we sought to investigate how well iPSC-derived neurons model the differential expression between adult human SNs and MNs. We compared single-nuclear RNA sequencing of dorsal root ganglia neuronal nuclei following neuronal nuclei antigen (NeuN) pull-down (Nguyen et al., 2021) with the annotated MN fraction of a recent spinal cord single-cell dataset (Yadav et al., 2023). Biological replicates were created by pooling all relevant single-cell expression data for each tissue donor to create “pseudobulk” samples.

**Figure 2:**
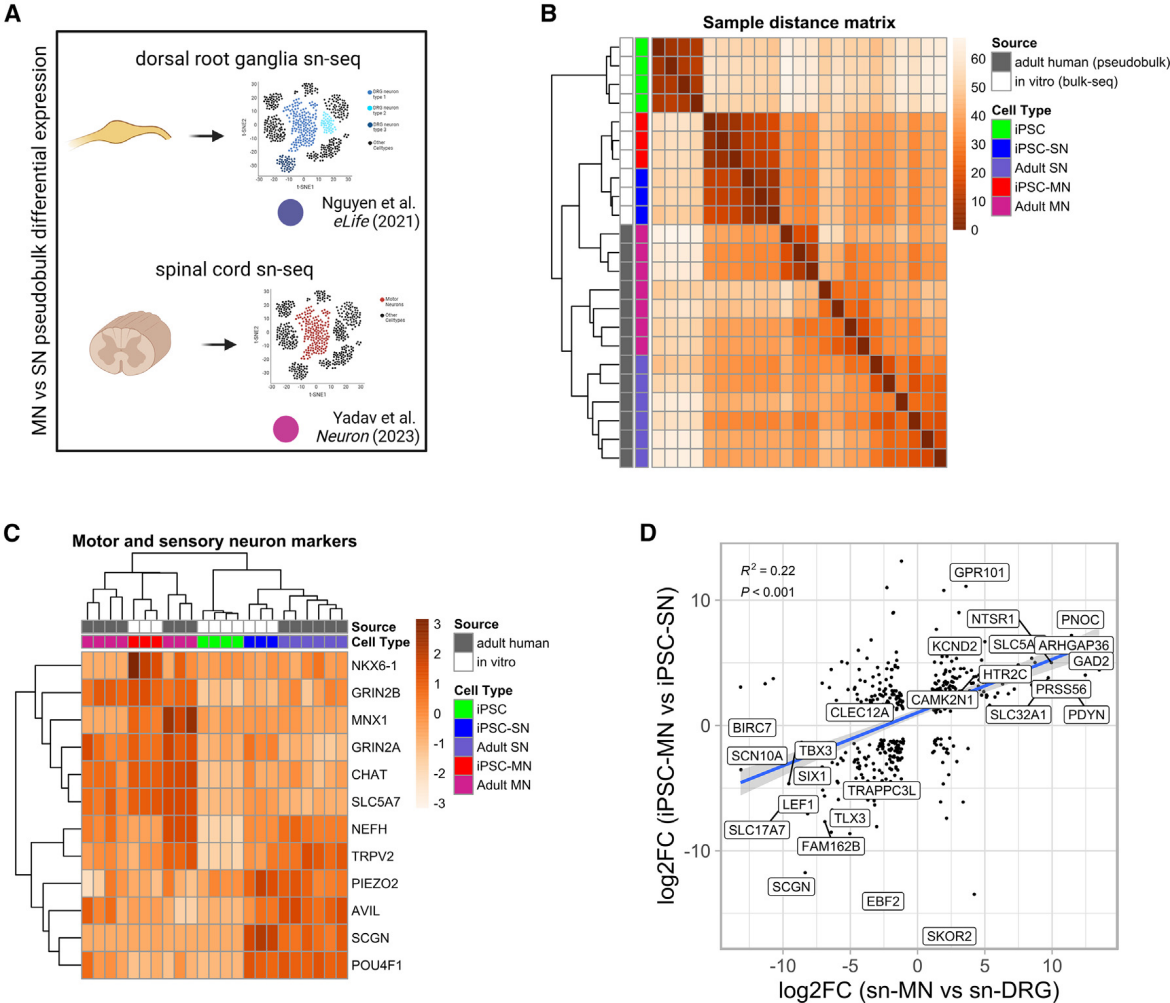
Comparison with single-cell adult datasets confirms relevance of iPSC model for studying cell-selective neuronal phenotypes but also reveals important differences (A) Experimental design showing sources for motor and sensory single-nucleus sequencing datasets used for pseudobulk differential expression. (B) Sample distance matrix that includes all datasets used in this study demonstrates hierarchical clustering of *in vitro* samples away from postmortem datasets but correct separation of motor and sensory neurons within each cluster. (C) Supervised clustering using heatmap of marker genes shows correct clustering of samples by cell type, independent of sample origin. The figure shows that some MN markers are consistent between iPSC MNs and postmortem samples but that there are also important differences between the model and adult tissue. (D) Scatterplot and linear correlation of differentially expressed genes between motor and sensory iPSC neurons versus differential expression between the pseudobulk adult datasets, demonstrate a significant correlation between the two comparisons.

The heatmap of the transformed sample distance matrix demonstrated clustering of the iPSC-derived samples away from the postmortem samples but with appropriate separation of cell types within these clusters (Figure 2B). Supervised hierarchical clustering of known SN and MN marker genes on the other hand yielded the expected clustering by cell type, indicating the presence of common cell type-specific features between iPSC-derived neurons and their adult counterparts (Figure 2C). Compared with adult MNs, iPSC MNs had similar expression of *CHAT*, *SLC5A7*, and N-methyl-D-aspartate (NMDA) receptor genes, but lower expression of *NEFH*, instead expressing developmental ventral neuronal cord transcription factor *NKX6.1*.

To specifically examine the utility of the iPSC model for the study of selective vulnerability, we assessed whether genes differentially expressed between motor and sensory iPSC-derived neurons were also differentially expressed in the postmortem dataset. For this, we plotted differentially expressed genes (false discovery rate [FDR] <0.05) above an absolute log_2_-fold threshold of 1 and found a significant correlation between the two comparisons (R^2^ = 0.22, Figure 2D). As the validity of MN clusters in single-cell spinal cord datasets is debated (Gautier et al., 2023), we performed the same analysis against a second adult dataset, created by comparing available laser capture microdissection RNA sequencing (LCM-seq) of pooled samples containing 120 captured MN nuclei from each donor (Nichterwitz et al., 2016), with an available RNA sequencing dataset of whole dorsal root ganglia (Ray et al., 2018), which yielded similar results (R^2^ = 0.17, Figure S2).

### Only a subset of antibody markers used to specify MNs and SNs can distinguish these cell types when derived from iPSCs

**Figure 3:**
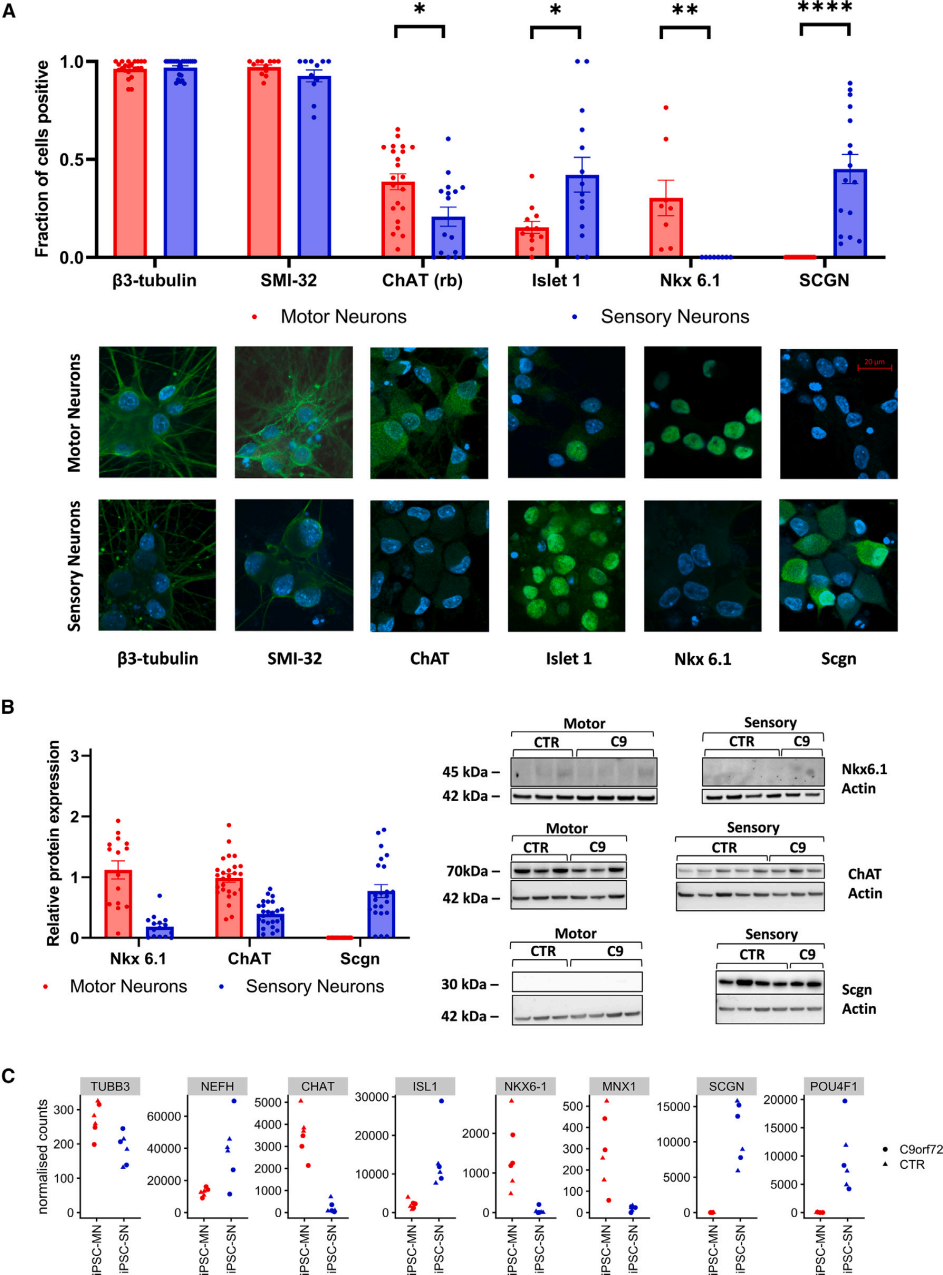
iPSC MNs and iPSC SNs can be distinguished by immunofluorescence and western blotting (A) Immunofluorescence imaging of iPSC MN and iPSC SN cultures five weeks after final plating, with representative images of the marker in each cell type below the bar graph. High proportion of β-3 tubulin- and SMI-32-positive cells confirms high efficiency and no difference in neurogenesis between both cultures. Nkx 6.1, only found iPSC MNs (*p* < 0.01), and secretagogin (Scgn), only found in iPSC SNs (*p* < 0.0001) confirm functional divergence of the two cultures. After setting of a threshold, higher levels of ChAT+ cells are seen in iPSC MNs compared to iPSC SNs (*p* < 0.05) and islet1+ cells were more frequent in iPSC SNs (*p* < 0.05). *n* = 2 differentiations. (B) Western blotting confirmed correct molecular weight and cell-type specificity of the Nkx 6.1 (*p* < 0.01) and secretagogin antibodies (*p* < 0.01), as well as the relative cell-type specificity of the ChAT antibody at the expected molecular weight of 70kDa (*p* < 0.05). *n* = 3 differentiations. (C) Gene expression of key motor and sensory neuron marker genes from RNA Sequencing data (derived from DESeq2 normalised counts matrix) confirms cell-type specificity and similar expression levels in *C9ORF72*+ and control neurons.

Despite specific expression of MN and SN markers linking iPSC neurons to their adult cell type, using immunofluorescence, we found that only two antibodies reproducibly distinguished iPSC MNs or iPSC SNs: Nkx6.1 was present only in iPSC MNs, and secretagogin, which is known to label the calcitonin gene-related peptide (CGRP) subtype of SNs, was solely present in iPSC SNs (Figure 3A). Unfortunately, we were not able to find an antibody for the pan-sensory neuronal marker Brn3α, as tested antibodies stained both iPSC MN and iPSC SN nuclei (Figures S3A and S3B).

The MN marker choline acetyltransferase (ChAT) is known to be expressed in some dorsal root ganglia cells and has diffuse cytoplasmic staining (rabbit monoclonal ChAT in Figure 3A; goat ChAT in Figure S3C). We used a fixed-threshold method, which demonstrated a difference in ChAT positivity between the two cell types. Finally, we were able to show that both iPSC MNs and iPSC SNs were positive for β3-tubulin and SMI-32, a heavy-chain neurofilament marker, indicating high neuronal purity (near 100%) following antimitotic treatment. As expected, islet1 was present in both cell types, but at a higher frequency in iPSC SNs, consistent with its differential gene expression in our cultures (*p* < 0.05, Figure 3A).

Western blot analysis on whole-culture lysates confirmed the immunofluorescence findings, with Nkx6.1 and secretagogin present in the relevant cell types, and ChAT being more abundant in iPSC MNs (*p* < 0.05, Figures 3B and S3C).

RNA sequencing data confirmed the presence of selective expression of the aforementioned genes, regardless of the *C9ORF72* HRE genotype (Figure 3C). Given the lack of a single standardized MN differentiation protocol, we also compared the gene expression profile of iPSC MNs in this study against three other published datasets of control iPSC MNs (Hall et al., 2017; Hawkins et al., 2022; Sommer et al., 2022), including one that reported >60% islet1-positive neurons. There was a high correlation with these datasets and with postmortem datasets (Figure S4A). Expression of MN markers was similar to that in other iPSC MN studies. Compared to all remaining studies, the iPSC MNs in this study had a particularly low expression of the proliferation marker *MKI67* (Figure S4B).

### MNs and SNs show similar expression of RNA foci and dipeptides

**Figure 4:**
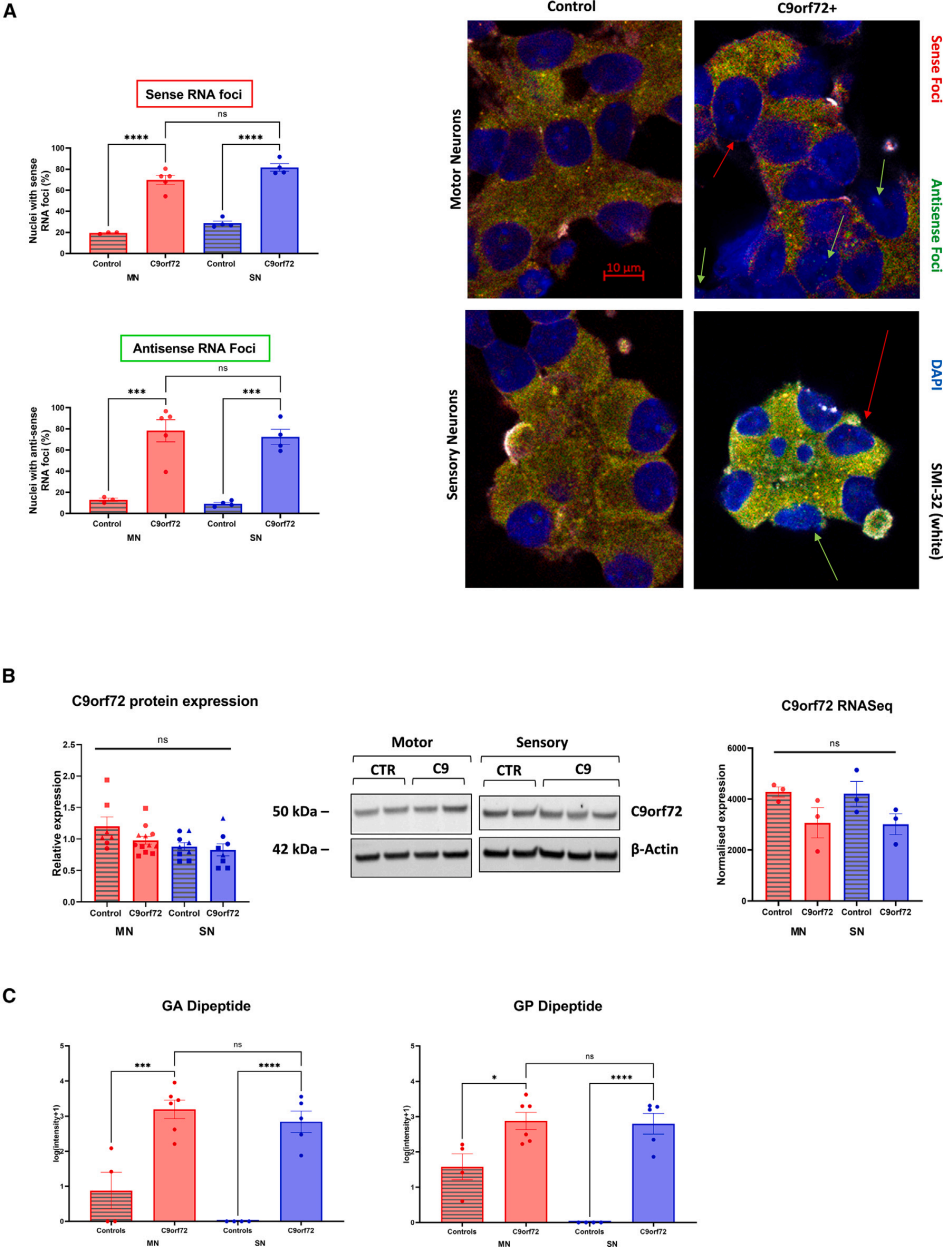
iPSC MNs and iPSC SNs from *C9ORF72*-positive patients have similar levels of RNA foci and dipeptide protein (A) RNA sense (*p* < 0.0001) and antisense (*p* < 0.01) foci were detected in both *C9ORF72*+ iPSC MNs and iPSC SNs, with detection of nonspecific background staining only in controls. No significant differences in the abundance of RNA foci were seen between the two cell types (*n* = 1 differentiation). (B) No significant differences in C9orf72 protein and *C9ORF72* mRNA expression in *C9ORF72*+ MNs or SNs (*n* = 3 differentiations, marked by different symbols). (C) ELISA quantification of GA and GP dipeptide protein in motor and sensory neurons. Analysis of pooled protein from up to 3 differentiations, 1 dot = 1 cell line.

Fluorescence *in situ* hybridization was performed for both sense and antisense RNA foci, which were found at similar frequencies in both iPSC SNs and iPSC MNs in *C9ORF72* HRE-positive lines (Figure 4A). No differences in C9orf72 protein or *C9ORF72* mRNA expression were seen in mutant iPSC MNs or iPSC SNs compared to controls, though a small difference would not have been captured by this study given its sample size (Figure 4B). An ELISA for poly-GP and poly-GA confirmed the presence of these dipeptides in both mutant iPSC SNs and iPSC MNs at similar levels (Figure 4C).

### iPSC MNs have fewer stress granules and a higher cytoplasmic/nuclear ratio of TDP-43 compared to iPSC SNs

To ascertain if reported iPSC MN phenotypes due to the *C9ORF72* HRE can be found in iPSC SNs, we investigated stress granule formation, TDP-43 distribution, survival, and expression of proapoptotic markers.

**Figure 5:**
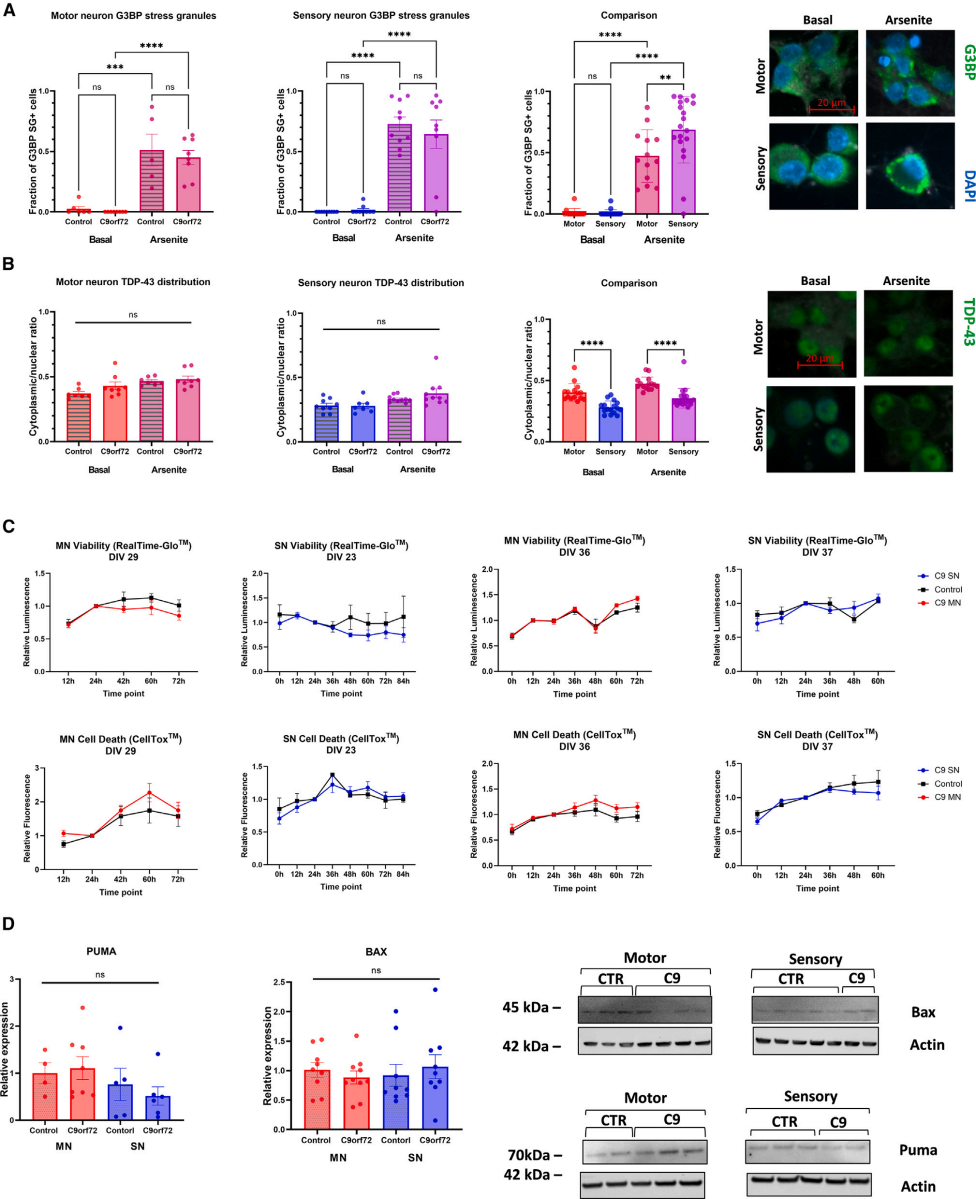
Lack of cellular phenotypes due to the *C9ORF72* HRE in iPSC SNs or iPSC MNs (A) No difference in arsenite-induced G3BP + stress granule (SG) assembly between controls and *C9ORF72*+ iPSC MNs or iPSC SNs. Independent of the mutation, iPSC SNs had a higher frequency of SGs following 1 h Arsenite stress than iPSC MNs. Stars denote significant differences between the basal and stressed conditions, *n* = 2 differentiations. (B) No difference between controls and *C9ORF72*+ iPSC MNs or iPSC SNs in either the basal condition or following 1 h of arsenite stress. Independent of the mutation, iPSC MNs had a higher ratio of cytoplasmic/nuclear TDP-43 staining in both the basal condition and after 1 h of arsenite stress. (C) No differences in viability (Real-Time Glo) or cell death (CellTox) between controls and *C9ORF72*+ iPSC MNs or iPSC SNs at both an early and late time point (*n* = 1 differentiations). (D) Quantification and representative images of staining for the proapoptotic markers PUMA and BAX, which did not show a difference between controls and *C9ORF72*+ iPSC MNs or iPSC SNs.

Ras-GTPase-activating protein binding protein (G3BP)-positive stress granules were rarely present under basal conditions but were highly abundant in response to arsenite stress, with no differences between control and *C9ORF72* HRE-positive cell lines in either iPSC MNs or iPSC SNs. However, iPSC SNs had more abundant arsenite-induced stress granules compared to iPSC MNs (Figure 5A). Results were independently replicated using poly(A)-binding protein (PABP) as an alternative marker (Figure S5A).

No TDP-43 puncta were observed in any cell lines, and there were no differences in nuclear/cytoplasmic staining intensity of TDP-43 between control and mutant cell lines in iPSC MNs and iPSC SNs under basal or stress conditions, though iPSC MNs had consistently higher relative cytoplasmic TDP-43 localization compared to iPSC SNs, under all conditions (Figure 5B).

Increased apoptosis and reduced survival have previously been reported in *C9ORF72* HRE-positive iPSC MNs (Abo-Rady et al., 2020; Shi et al., 2018). We performed longitudinal cell viability and cell death assays using the RealTime Glo and CellTox assays following treatment with 0.5 mM sodium arsenite for 1 h, and this did not reveal a difference between *C9ORF72* HRE-positive cell lines and controls in either iPSC MNs or iPSC SNs at two different culture time points (Figure 5C). Similarly, we could not detect cleaved caspase 3 or changes in the relative expression of the proapoptotic proteins Bax and Puma in control or *C9ORF72* HRE-positive iPSC MNs and SNs even after exposure to arsenite (Figures S5B and S5D).

### C9ORF72 HRE results in specific slowing of retrograde lysosomal transport and bidirectional slowing of mitochondrial transport in both MNs and SNs

**Figure 6:**
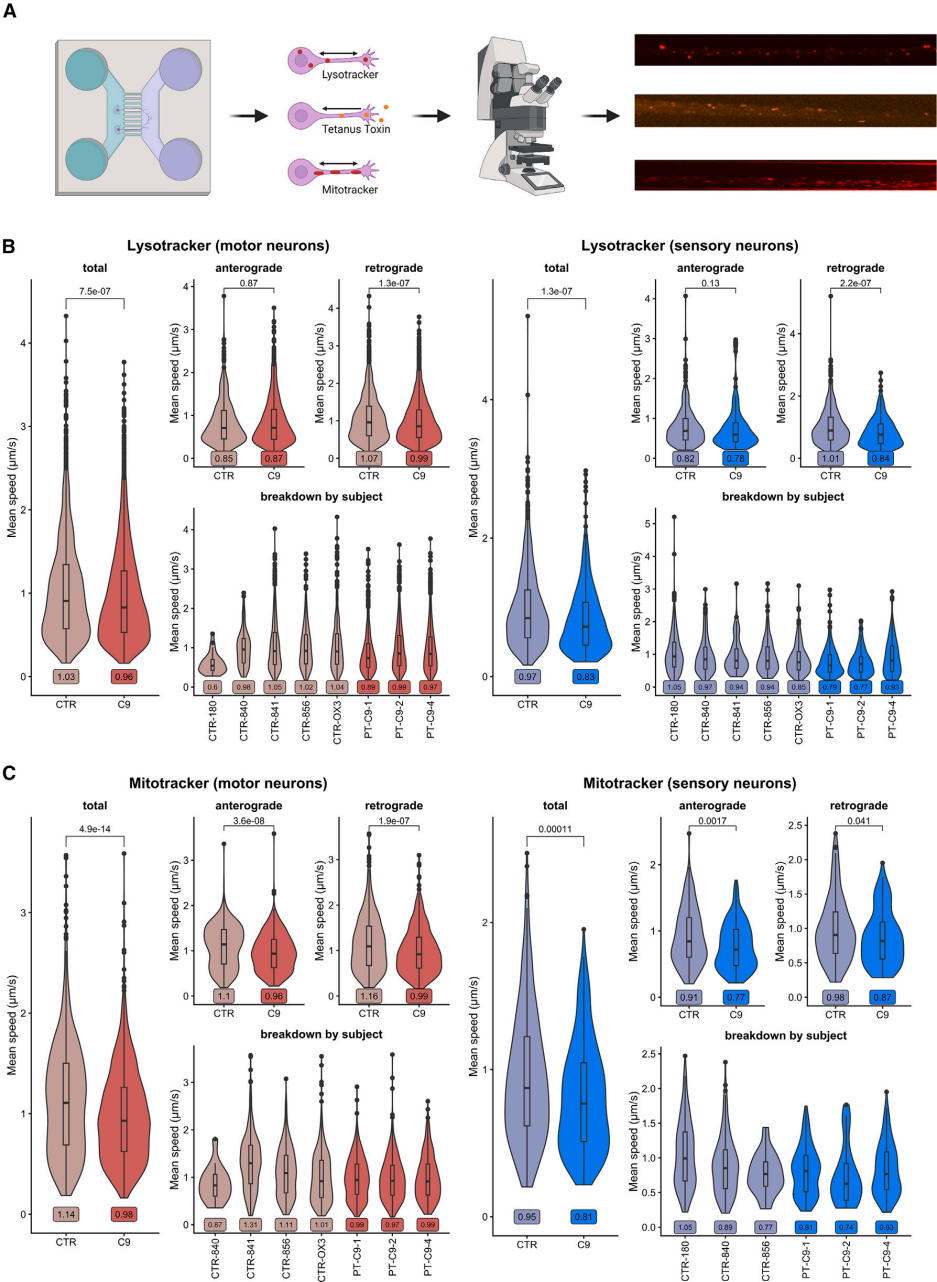
Specific slowing of endosomal transport and mitochondrial transport due to the *C9ORF72* HRE is seen equally in iPSC MNs and iPSC SNs (A) Schematic showing a typical microfluidic chamber and the experimental setup with three fluorophores for imaging lysosomal, retrograde endosomal, and mitochondrial transport, including example still images from the respective movies. (B) Results from LysoTracker analysis in iPSC MNs (red) and iPSC SNs (blue) showing slowing of lysosomal transport in both *C9ORF72*+ iPSC MNs and *C9ORF72*+ iPSC SNs compared to controls, which is seen only in retrograde and not anterograde transport. Lower panel shows breakdown between lines to give indication of biological variability. Violin plots with boxplot insert showing median, with mean indicated in the label below. *n* = 3 differentiations. (C) Results from MitoTracker analysis in iPSC MNs (red) and iPSC SNs (blue) showing slowing of mitochondrial transport in both *C9ORF72*+ iPSC MNs and *C9ORF72*+ iPSC SNs compared to controls, which is seen in both retrograde and anterograde transport. Lower panel shows breakdown between lines to give indication of biological variability. Violin plots with boxplot insert showing median, with mean indicated in the label below. *n* = 3 differentiations.

As the axon is the distinguishing feature of neurons and could be a site of early neuronal pathology in ALS, based on evidence from genetic studies (Zhang et al., 2022), animal models (Sleigh et al., 2020; Williamson and Cleveland, 1999), and previous reports in *C9ORF72* HRE-positive iPSC MNs (Dafinca et al., 2020; Mehta et al., 2021), we sought to assess multiple modalities of transport using custom-made microfluidic devices. Samples were assessed at 21 days following the final seeding of neurons. We used LysoTracker to assess both anterograde and retrograde lysosomal transport and fluorescently labeled tetanus toxin (HcT), which preferentially labels retrograde endosomal transport. Finally, we also used MitoTracker to assess mitochondrial transport. Samples were analyzed using Difference Tracker, and image processing and analysis were fully automated (Figure 6A).

A total of 72,959 particles were identified, of which 33,551 passed quality control (non-stationary; all particles moving horizontally in the video). A total of 15,639 LysoTracker particles, 12,609 tetanus toxin particles, and 5,303 MitoTracker particles were analyzed. A reduction in the mean velocity of travel was observed in lysosomal transport in both iPSC MNs and SNs with the *C9ORF72* HRE compared to controls (p < 1e−06), and this was specific for retrograde but not anterograde transport in both cell types (Figure 6B). Retrograde transport of tetanus toxin was not affected in either cell type (Figure S6A). Using MitoTracker, we observed a slowing of mitochondrial transport due to the *C9ORF72* HRE by approximately 15% in both iPSC MN and SNs with the *C9ORF72* HRE (*p* < 0.001), and this was seen in both anterograde and retrograde direction in both cell types.

More detailed analysis of the characteristics of travel did not point to a specific mechanism for the axonal slowing observed in this study, as it was accompanied by both an increased proportion of time of particles spent in pause and slowing of maximal velocity in all relevant MitoTracker and LysoTracker experiments (Figure S6B).

## Discussion

In this study, we carried out a detailed phenotypic characterization of iPSC MNs and SNs from patients carrying the *C9ORF72* HRE, with the aim of finding MN-specific phenotypes that could give rise to selective vulnerability. We provide evidence for similar levels of RNA sense and antisense foci as well as GA and GP dipeptide production in iPSC MNs and SNs. Both RNA foci and dipeptide have been postulated to be potential gain-of-function mechanisms by which the *C9ORF72* HRE could disturb cellular homeostasis, eventually resulting in ALS. In this study we were not able to replicate differences in stress granule formation and TDP-43 distribution or survival after arsenite stress due to the *C9ORF72* HRE. We then demonstrate that deficits in retrograde lysosomal and bidirectional mitochondrial transport in iPSC-derived neurons from ALS patients with the *C9ORF72* HRE are not MN specific but that matching deficits can also be observed in iPSC SNs from the same patients. Given that SNs are relatively spared in ALS, this suggests that changes in axonal transport due to the *C9ORF72* HRE observed here and in previous studies are unlikely to be responsible for selective vulnerability in MNs in isolation. A contribution of *C9ORF72* HRE-dependent slowing of axonal transport to the disease in the context of an aging nervous system with its complex cellular connections and in combination with other pathological processes cannot be excluded.

The mechanism of how the *C9ORF72* HRE causes axonal transport abnormalities remains under investigation. The specificity of deficits only in retrograde lysosomal transport and bidirectional mitochondrial transport, with sparing of endosomal transport and lysosomal anterograde transport, suggests that axonal degeneration through general failure of microtubule homeostasis is unlikely. One *in vitro* study suggested that the presence of arginine-containing dipeptides causes the mitochondrial transport machinery to stall (Fumagalli et al., 2021). Another study argued that abnormal mitochondrial bioenergetics may result in the slowing of mitochondrial transport (Mehta et al., 2021), although we could not replicate the differential expression of mitochondrially encoded genes reported in that study. The very low number of differentially expressed genes in our study indicates that both the mutation and its axonal phenotype are relatively well tolerated in the developing MN and that the axonal transport deficits are not linked to the pathogenic variant through changes in transcriptional regulation.

In this study, we were not able to replicate abnormalities of cellular homeostasis that have been reported in studies of *C9ORF72* HRE iPSC MNs previously (Braems et al., 2020). Of note, a number of these findings were reported by our research group previously (Dafinca et al., 2016, 2020). Compared to previous studies, this study is characterized by longer time in culture, enabled by changes in the adhesion layer and the addition of an antimitotic treatment, which successfully removed contaminating non-neuronal dividing cells and reduced overall variance. We have not used stressors other than sodium arsenite in this study, and it is possible that prolonged or different stress conditions could result in a loss of cellular homeostasis due to the *C9ORF72* HRE in the lines presented here.

The absence of widespread metabolic disturbances is supported by lack of differential expression due to the *C9ORF72* HRE, independently replicated in both iPSC MNs and iPSC SNs. While our sample size is small, it argues against substantial metabolic disturbance in C9orf72 at basal conditions, in line with a recent large-scale transcriptomic study which compared over 300 patient-derived iPSC-derived MN lines with controls, which included 29 lines from *C9ORF72* HRE carriers, and did not find significant transcriptional derangements (Workman et al., 2023). In contrast to this prospective study, a meta-analysis of published smaller sequencing studies has found an apoptotic signal (Ziff et al., 2023), which may potentially indicate the presence of publication bias toward stronger phenotypes in existing smaller studies.

The presence of the *C9ORF72* HRE mutation is not a sufficient condition for the development of ALS, which occurs in adult MNs in the context of aging and a complex multicellular nervous system. This study demonstrates that iPSC-derived MNs and SNs have divergent identities and recapitulate some of the features of the adult cell type but also emphasizes differences. These may partly be explained by important limitations of the adult datasets used in this study, including the nuclear origin of the RNA in single-cell studies, the high glial component in both the single-cell and the laser-captured MNs, and the variability of postmortem datasets. Notwithstanding these inconsistencies, iPSC MNs remain transcriptomically distinct from their adult counterparts and may therefore potentially lack the characteristics required for ALS to manifest.

The study has two implications for the understanding of ALS modeling using iPSCs. Firstly, differential expression changes due to the ALS-causing *C9ORF72* HRE variant in iPSC-derived MNs are small compared to the differences between iPSC MNs and healthy adult MNs, and therefore any transcriptomic changes in the model may not be simply translatable to postmortem studies. Secondly, at least some *in vitro* phenotypes seen in iPSC-derived MNs with the *C9ORF72* HRE may be equally present in cell types that do not degenerate in ALS and therefore less likely to be primary drivers of cell-specific neurodegeneration.

The findings in this study raise important considerations for future investigations of the *C9ORF72* HRE. Understanding how to further encourage maturation of iPSC MNs toward the adult MN type will be crucial in neurodegenerative disease and may necessitate increasing the complexity of the model through functional stimulation or the addition of other cell types, which will require stringent control of experimental variability. The lack of a strong transcriptomic disease signature in *C9ORF72* HRE iPSC neurons is an important challenge in this context and will require further detailed study of different stress paradigms, including chronic stress and stress recovery, and may require more detailed methods than whole-cell quantification of mRNA and protein to capture subtle differences of cellular homeostasis.

In conclusion, this study demonstrates how the ability of iPSCs to generate divergent cell types can be harnessed to better understand cell-type specificity in ALS. It provides evidence that the axonal phenotypes reported in iPSC MNs are also present in iPSC SNs and therefore are unlikely to be the sole driver of selective vulnerability. Future work, both in terms of developing the iPSC model systems and understanding the differences between adult MNs and SNs, will be required to understand the basis for selective vulnerability in ALS.

## Experimental procedures

### Resource availability

#### Lead contact

Further information and requests for resources and reagents should be directed to and will be fulfilled by the lead contact, Dr. Jakub Scaber (Jakub.scaber@ndcn.ox.ac.uk).

#### Materials availability

This study did not generate new unique reagents.

#### Data and code availability

The data generated in this study have been uploaded to the Gene Expression Omnibus repository GSE262929. The code used for the generation of RNA sequencing figures and for the imaging analysis is available at https://github.com/jscaber/sensorimotor.

### Use of human-derived iPSCs in this study

Previously published iPSC lines from three patients (two clones from each patient), as well as five sex- and age-matched controls were used (Ababneh et al., 2020; Dafinca et al., 2016, 2020) and are summarized in Table S1. iPSC lines were derived from human skin biopsy fibroblasts collected under ethical approval granted by the South Wales Research Ethics Committee (WA/12/0186) and the South-Central Berkshire Research Ethics Committee (REC10/H0505/71) in the James Martin Stem Cell Facility, University of Oxford, under standardized protocols. Cells were passaged using EDTA (0.5 mM), expanded to produce consistent, frozen cell stocks for the study, and tested negative for mycoplasma using the MycoAlert mycoplasma detection kit (Lonza).

### MN and SN differentiation

MN differentiation (Ababneh et al., 2020) and SN differentiation (Clark et al., 2021) followed previously published protocols with minor modifications, most notably including the addition of cytosine arabinoside in the MN differentiation and coating with polyethyleneimine and Geltrex at the final plating for both differentiations. The day of induction was defined as DIV 0. Detailed methods are available in the , and a schematic representation can be found in Figure 1A. Cells were harvested five weeks after final plating, which was DIV 45 for iPSC SNs and DIV 52 for iPSC MNs for all experiments except where specified. Growth factors used can be found in Table S4.

### Protein extraction, quantification, and western blotting

Protein extraction, quantification using the bicinchoninic assay, and western blotting were carried out using standard laboratory practices. A total of three differentiations were performed. Across blots, data were normalized against the mean of MN controls on each blot. Detailed methods including antibodies used are available in the supplemental information.

### Immunocytochemistry and fluorescence *in situ* hybridization

Cells were grown on glass coverslips and fixed five weeks after plating. Cells conditions were treated with 0.5 mM sodium arsenite 1 h prior to fixation and staining in relevant experiments. Immunocytochemistry was carried out using standard laboratory practices. Fluorescence *in situ* hybridization was carried according to previously published methods (Ababneh et al., 2020). Detailed methods including antibodies used are available in the supplemental information.

### Live imaging of axonal transport using microfluidic devices

Microfluidic devices were produced according to a previously described protocol (Park et al., 2006) with detailed methods in the supplemental information. Channels were coated with Geltrex for at least 1 h before plating of ∼25,000 neurons. During the duration of culture in the devices, a gradient was maintained with 50 μL additional medium and a 5-fold concentration of growth factors in the axonal wells compared to the cell body side.

Neurons were grown in microfluidic devices for 3 weeks after final seeding (DIV 32 for iPSC SNs, DIV 39 for iPSC MNs), as longer cultures resulted in degradation of the axons. Fluorescent molecular dyes were added to the medium 45 min prior to imaging. Separate axonal chambers were used for each dye, and chambers were never reused. LysoTracker Red DND-99 and MitoTracker Red CMXRos at concentrations of 70 nM were applied to both sides. Tetanus toxin was added to the axonal side only at a concentration of 40 nM. Microfluidic chambers were imaged in a Zeiss scanning disc confocal microscope with an incubation chamber containing 5% CO_2_ and heated to 37°C. Imaging was performed using a 63x apochromat lens, on a single plane (to minimize bleaching and delay) with an image of the distal axonal groove taken every 2 s for a total of 10 min per experiment.

### Live image analysis

Live image analysis was performed using the software package Difference Tracker (Andrews et al., 2010), which was automated using a custom Python script in Fiji (Schindelin et al., 2012). The raw output from the software was extracted and processed in R using a custom script, which added features such as velocity/direction and pause time analysis.

### Dipeptide repeat protein extraction and ELISA

Frozen pellets were lysed in buffer and processed as described previously (Vahsen et al., 2023), and detailed methods are available in the supplemental information.

### Confocal imaging and image analysis

Images of markers of cell type and cell stress were obtained at 63x resolution using a Zeiss spinning disk confocal microscope and analyzed using Fiji. Nuclear stains (islet 1, Nkx 6.1) were counted if the nuclear staining was stronger than the cytoplasmic staining. For ChAT and secretagogin, an arbitrary fixed fluorescence intensity threshold was selected for each differentiation, such that no staining was present in non-neuronal cells.

PABP and G3BP were quantified manually by counting the number of cells containing 1 or more bright PABP/G3BP + puncta 0.1–4 μm in diameter (Mahboubi and Stochaj, 2017). The ratio of cytosol/nucleus fluorescence intensity was used as a measure of mislocalization. Images of RNA foci were obtained at 63x using Zeiss LSM 780 and were analyzed in individual slices of the confocal z stack, counting the number of foci per DAPI-stained nucleus.

### Viability and toxicity assays

We performed a longitudinal survival and toxicity assay by using the Real-Time Glo MT viability assay (Promega) and the CellTox green cytotoxicity assay (Promega) and performed this at an early time point (DIV 23 in iPSC SNs and DIV 29 in iPSC MNs) and a late time point (DIV 36 in iPSC SNs and DIV 37 in iPSC MNs), taking longitudinal readings every 12–24 h up to 72 h. Data were normalized to the 24-h readout.

### RNA sequencing and acquisition of external sequencing data

RNA extraction from iPSC MNs and iPSC SNs was performed using the QIAGEN RNeasy micro kit 5 weeks after final plating from the control cell lines 180, 840, and 856 (both cell types) and the patient lines C9-02-03, C9-04-12 (both cell types), C9-01-06 (MNs), and C9-01-07 (SNs). RNA abundance and quality were assessed using NanoDrop and the Bioanalyzer nano RNA kit. Library preparation was done using paired-end sequencing with a read length of 150 bp. Sequencing was performed on the Illumina NovaSeq 6000. External sequencing datasets were obtained from the Gene Expression Omnibus (GEO) with accession numbers GSE144208, GSE98288 (Hall et al., 2017), GSE201407 (Sommer et al., 2022), GSE203168 (Hawkins et al., 2022), GSE190442 (Yadav et al., 2023), GSE168243 (Nguyen et al., 2021), and GSE76514 (Nichterwitz et al., 2016) or from the dbGaP web site, under phs001158.v2.p1 (Ray et al., 2018). For the single-nucleus spinal cord dataset (GSE190442), the bam data were filtered using the experimental matrix for barcodes assigned to MNs and then converted back to fastq format using samtools.

### Sequencing analysis

RNA sequencing analysis was performed using automated pipelines using the cgat-developers software (Cribbs et al., 2019), available at https://github.com/cgat-developers/cgat-flow/ (branch JS-exome). Mapping against Ensembl genome version 101 was performed with STAR using the 2-pass method using a standard hg38 genome, with the following options on first pass: “--alignIntronMin 20 --alignIntronMax 1000000 --alignMatesGapMax 1000000 --alignSJoverhangMin 8 --outFilterMismatchNoverReadLmax 0.04 --outFilterScoreMinOverLread 0.9 --outFilterMatchNminOverLread 0.9”. Count tables were obtained using featureCounts for the coding subset of the gene set only (Liao et al., 2014).

Exploratory and differential expression analysis was carried out in R. Differential expression analysis was performed using DESeq2 (Love et al., 2014), with a false discovery threshold of 0.05, and using the model ∼group+sex. Log-fold change shrinkage was performed using apeglm (Zhu et al., 2019). GSEA was performed using fgsea (Sergushichev, 2016), and overrepresentation analysis was performed using goseq (Young et al., 2010). Expression correlation was performed using vidger.

### Other analyses and illustrations

Analyses for Figures 3, 4, 5, S1, and S3 were produced in GraphPad Prism v9. Comparisons of two groups were performed by two-tailed unpaired t tests and multiple group comparisons by one-way ANOVA with appropriate *post hoc* tests as indicated in the figure legends. Data are presented as single data points and means ± SEM. Differences were considered significant when *p* < 0.05 (^∗^*p* < 0.05; ^∗∗^*p* < 0.01; ^∗∗∗^*p* < 0.001; ^∗∗∗∗^*p* < 0.0001; ns: not significant). Illustrations were created with BioRender.com (Figures 2A and 6A) and Microsoft PowerPoint (Figure 1A).

## Supplemental information

Document S1. Figures S1‒S6 and Tables S1‒S4

Document S2. Article plus supplemental information
