# Supplementary material for: Cellular and axonal transport phenotypes due to the C9ORF72 HRE in iPSC motor and sensory neurons
Source: Stem Cell Reports. 2024 Jun 13;19(7):957–72. doi: 10.1016/j.stemcr.2024.05.008 (PMC11252479; doi:10.1016/j.stemcr.2024.05.008)
Supplement: Document S1. Figures S1‒S6 and Tables S1‒S4 [file mmc1.pdf]

Stem Cell Reports, Volume *19*

Supplemental Information

Cellular and axonal transport phenotypes due to the *C9ORF72* HRE in iPSC motor and sensory neurons

Jakub Scaber, Iona Thomas-Wright, Alex J. Clark, Yinyan Xu, Björn F. Vahsen, Mireia Carcolé, Ruxandra Dafinca, Lucy Farrimond, Adrian M. Isaacs, David L. Bennett, and Kevin Talbot

Supplementary Tables

iPSC line	abbrev.	sex	age	ALS variant	PMID
OX3-06	OX3	male	49	-	29604226
SFC180-03-03	180	female	60	-	28827786
SFC840-03-03	840	female	67	-	26905200
SFC841-03-01	841	male	36	-	27097283
SFC856-03-04	856	female	78	-	28827786
C9-01-07<br>C9-01-06	C9-1	male	72	*C9ORF72*<br>(~970 G_4C_2 repeats)	32330447
C9-02-02<br>C9-02-03	C9-2	female	58	*C9ORF72*<br>(~1000 G_4C_2 repeats)	27097283
C9-04-12<br>C9-04-01	C9-3	male	39	*C9ORF72*<br>(>500 G_4C_2 repeats)	32330447

Supplementary Table 1: iPSC lines used in this study. Clones from the same patient are grouped. Abbreviations: PMID = PubMed identification number; ALS = amyotrophic lateral sclerosis

Antibody	Species	Dilution	Source	Catalogue number
β-Actin	Ms	1:10000 WB	Invitrogen	AM4302
β-Actin	Rb	1:10000 WB	Abcam	Ab227387
Advillin/p92	Rb	1:1000 WB	Abcam	Ab72210
Bax	Rb	1:1000 WB	Abcam	Ab32503
Brn3a	Rb	1:500 WB	Merck	MAB5945
Brn3a	Ms	1:100 WB	Merck	MAB1585
C9orf72	Ms	1:500 WB	GeneTex	GTX632041
Caspase 3	Rb	1:1000 WB	Cell Signalling Technology	9662S
ChAT	Gt	1:1000 WB<br>1:100 IF	Merck	AB144P
ChAT	Rb	1:1000 WB<br>1:1000 IF	Abcam	ab178850
Cleaved Caspase-3	Rb	1:1000 IF<br>1:1000 WB	Cell Signalling Technology	9661S
G3BP	Ms	1:1000 IF	Abcam	ab56574
Hb9	Ms	1:1000 WB	DSHB	81.5C10-s
Islet-1	Ms	1:1000 WB	DSHB	40.206
Nkx6.1	Ms	1:300 WB<br>1:200 IF	DSHB	F55A12
PABP	Rb	1:500 IF	Abcam	ab21060
PUMA	Rb	1:1000 WB	Proteintech	55120-1-AP
TDP-43	Rb	1:200 IF	ProteinTech	10782-2-AP
Tuj1	Rb	1:1000 IF	Abcam	ab15568
Tuj1	Ch	1:1000 IF	Abcam	ab107216

Supplementary Table 2: Primary antibodies used for immunofluorescence and western blotting. Abbreviations: Ch = chicken; Gt = goat; IF = immunocytochemistry; Ms = Mouse; Rb = Rabbit; WB = western blotting.

Antibody	Species	Dilution	Source	Catalogue number
Anti-rabbit AlexaFluor647	Dy	1:1000 IF	Invitrogen	A31573
Anti-rabbit AlexaFluor546	Dy	1:1000 IF	Invitrogen	A10040
Anti-rabbit AlexaFluor488	Dy	1:1000 IF	Invitrogen	A21206
Anti-chicken AlexaFluor647	Gt	1:1000 IF	Invitrogen	A21449
Anti-mouse AlexaFluor568	Gt	1:1000 IF	Invitrogen	A10037
Anti-mouse AlexaFluor488	Gt	1:0000 IF	Invitrogen	A11001
Anti-guineapig AlexaFluor568	Gt	1:1000 IF	Invitrogen	A11075
Anti-goat AlexaFluor647	Dy	1:1000 IF	Invitrogen	A21447
Anti-mouse IgG	Dy	1:5000 WB	LI-COR	926-32212
Anti-mouse IgG HRP	Sh	1:5000 WB	Amersham	NA934V
Anti-rabbit IgG	Dy	1:5000 WB	LI-COR	926-68073
Anti-rabbit IgG HRP	Dy	1:5000 WB	Amersham	NA931V
Anti-goat IgG HRP	Dy	1:5000 WB	Invitrogen	PA1-28664

Supplementary Table 3: Secondary antibodies used for immunofluorescence and western blotting. Abbreviations: Dy = donkey; Gt = goat; HRP = horseradish peroxidase; IF = immunocytochemistry; Sh = Sheep; WB = western blotting.

Growth Factor	Source	Catalogue number
Glial-Derived Neurotrophic Factor (GDNF)	Preprotech	450-10
Brain Derived Neurotrophic Factor (BDNF)	Preprotech	450-02
beta-Nerve Growth Factor (NGF)	Preprotech	450-01
Neurotrophin-3 (NT-3)	Preprotech	450-03

Supplementary Table 4: Growth factors used during differentiation.

Supplementary Methods

Motor neuron differentiation

Cryopreserved iPSCs were plated onto Geltrex and grown in mTeSR1 (StemCell Technologies) until 80% confluency, at which timepoint the differentiation was induced (DIV 0). Throughout the differentiation cells were maintained in basal media containing Dulbecco's Modified Eagle's Medium/nutrient mixture F-12 (DMEM/F-12) and Neurobasal medium in a 1:1 ratio, supplemented with 2% B27, 1% N2, ascorbic acid (0.5μM), Anti-Anti and β-mercaptoethanol (50nM) (all ThermoFisher). On DIV 0-3, wnt pathway agonist Chir99021 (3μM; Tocris Bioscience) and AMPK antagonist Compound C (1μM; Merck) were present to induce spinal MN progenitors. Retinoic acid (RA, 1μM; Sigma) and smoothened agonist (SAG, 500nM; Tocris Bioscience) were added on DIV 2. Between DIV 0 and 9 media changes (100%) were performed daily. On DIV 9, cells were passaged 1:3 using Accutase (ThermoFisher), and then fed 3 times per week with basal medium supplemented with RA and SAG.

On DIV 18, cells were dissociated with accutase, counted using trypan blue, and replated onto 0.07% polyethyleneimine (PEI; Merck)/Geltrex. A density of ~1,000,000 cells per well was used for 6-well plates and ~100,000 cells per well for 24-well plates. Basal media was supplemented with rock inhibitor, and γ-secretase inhibitor DAPT (10μM; Tocris Bioscience) was added to promote maturation. Media also contained growth factors BDNF and GDNF (both 10ng/ml; PreproTech). On DIV 19 cytosine arabinoside (AraC, 1μM; Sigma) was added. On DIV 23 AraC and DAPT were removed and mouse laminin (0.5μg/ml; ThermoFisher) was added for subsequent three times weekly 50% media changes. Motor neuron pellets and coverslips were collected five weeks after final plating (DIV 53). A schematic of the differentiation can be found in Figure 1A.

Sensory Neuron Differentiation

Cryopreserved iPSCs were thawed and grown in mTeSR until ~80% confluent. The sensory neuron differentiation followed a previously published protocol[23], by switching to KSR media (KnockOut™ DMEM/F-12, 15% KnockOut Serum Replacement; 1% GlutaMAX, 1% non-essential amino acids, 100μM β-mercaptoethanol and 1% Anti-Anti) containing SMAD inhibitors SB431542 (10μM; Sigma) and LDN-193189 (100nM; Sigma). On DIV 2 Chir99021 (3μM), DAPT (10μM) and FGF signalling inhibitor SU5402 (10μM; Sigma) was added. On DIV 6 dual SMAD inhibition was withdrawn. Between DIV 4 and 10 basal media was switched in 25% increments from KSR media to N2 Media (Neurobasal media, 2% B27 supplement, 1% N2 supplement, 1% GlutaMAX and 1% Anti-Anti). From DIV 0-11 media changes (100%) were performed daily.

On DIV 11 cells were dissociated with TrypLE (ThermoFisher), counted and replated onto PEI/Geltrex coated plates as described for MNs. SU and DAPT were removed and media was supplemented with growth factors NGF (25ng/ml; Peprotech), BDNF (25ng/ml), GDNF (25ng/ml) and Neurotrophin-3 (25ng/ml; Peprotech). As for MNs, rock inhibitor was added on the DIV of splitting and AraC was present between DIV 11 and 13. On DIV 13 AraC was

removed and laminin was added to the media. From DIV 13 to media changes (50%) were performed three times a week. From around DIV 30 the concentration of all four growth factors was reduced to 10ng/ml. Sensory neuron pellets and coverslips were collected five weeks after final plating (DIV 45). A schematic of the differentiation can be found in Figure 1A.

Protein extraction and Quantification

After a wash with PBS, cells were collected in a small volume of phosphate buffer saline (PBS) and pelleted at 3000rpm for 5 minutes at 4°C. Cells were lysed in RIPA buffer (Sigma) and protease inhibitors (Roche) following mechanically homogenisation. After 30 minutes lysates were centrifuged at 10,000rpm for 5 minutes at 4°C and the pellets were discarded. Total protein concentration of the supernatant was determined using the bicinchoninic acid assay (Sigma) according to the manufacturer's instructions.

Western blotting

For gel electrophoresis, protein in RIPA buffer was mixed with NuPAGE LDS sample buffer and NuPAGE sample reducing agent and heated to 95°C for 5 minutes, except where indicated. 10µg per well was loaded on NuPAGE 4-12% Bis-Tris gels (ThermoFisher) and then run at 100V for 120 minutes. Resolved protein was transferred to nitrocellulose membranes using the iBlot 2 Gel Transfer Device (ThermoFisher). Blots were blocked for 1 hour at room temperature in Tris-buffered saline (TBS) containing 0.1% Tween-20 and 5% skimmed milk and incubated overnight at 4°C with primary antibodies (Table 2) in TBS + 0.1% Tween-20 + 1% skimmed milk. Blots were washed three times and incubated with secondary antibody (Supplementary Table 3) in TBS + 0.1% Tween-20 + 1% skimmed milk for 1 hour at room temperature. Blots were washed a further three times before visualisation on a Bio-Rad ChemiDoc. Integrated optical density of the protein bands was measured using Fiji and normalised to β-actin for the same sample.

Immunocytochemistry

Cells grown on glass coverslips were fixed in 4% methanol-free paraformaldehyde for 30 minutes and washed three times with PBS. Coverslips were blocked and permeabilised (0.1% Triton-X; Dow, and 5% donkey serum; BioRad, in PBS) for 1 hour and then incubated overnight with primary antibody in antibody buffer (0.1% Triton-X and 1% donkey serum in PBS). Samples were rinsed in wash buffer (0.1% Triton-X in PBS) for ten minutes and then incubated with secondary antibodies in antibody buffer for one hour. Coverslips were washed again and then incubated with 4',6-Diamidino-2-Phenylindole (DAPI, 1:10,000 dilution in PBS) for ten minutes, followed by a PBS-only wash. Coverslips were mounted in Prolong Diamond Antifade mountant (ThermoFisher).

DPR extraction and ELISA

Frozen cell pellets were lysed using RIPA buffer containing an EDTA-free protease inhibitor cocktail (2x, Sigma) and 10% sodium dodecyl sulphate (SDS, ThermoFisher) and sonicated at 30% AMP for 3 x 10 seconds with 5 s-intervals. The probe was cleaned with ethanol between samples. Samples were centrifuged at 17,000xg for 20 minutes at 16°C and the pellets were discarded. Total protein

concentration of the supernatant was determined using a BCA assay and all samples were adjusted to 0.5mg/ml. Plates were coated with unlabelled anti-poly (GP) or anti-poly (GA) antibodies. After blocking, samples were loaded at 45 µg of protein per well for poly (GP) and 27 µg for poly (GA). Biotinylated anti-poly (GP) and anti-poly (GA) antibodies were used as detectors, followed by sulfo-tagged streptavidin (Meso Scale Discovery, R32AD). Plates were read with the MSD reading buffer (Meso Scale Discovery, R92TC) using the MSD Sector Imager 2400. Signals correspond to intensity of emitted light upon electrochemical stimulation of the assay plate. Prior to analysis, the average reading from a calibrator containing no peptide was subtracted from each reading.

Fluorescence in situ hybridisation

MNs and SNs were fixed in 4% paraformaldehyde for 30 minutes and washed three times with PBS. All work was carried out in an RNase free environment, using RNase ZAP (ThermoFisher). Cells were permeabilised with 0.1% Triton-X and then washed three times in PBS. Tissue was dehydrated with serial addition of 70%, 90% and 100% ethanol and then allowed to air dry before rehydration in PBS followed by 2x Standard Sodium Citrate (SSC) buffer (Sigma). Coverslips were incubated at 80°C for 45 minutes with pre-hybridisation solution (50% Formamide; ThermoFisher and 2x SSC in distilled water) followed by two hours in hybridisation solution (2x SSC, 0.016% BSA; ThermoFisher, 0.8mg/ml salmon sperm DNA; ThermoFisher, 0.8 mg/ml yeast tRNA; ThermoFisher, 8% Dextran sulphate; Amresco, 50% Formamide, 1.6 mM Vanadyl Ribonucleotide; BioLabs, 5mM EDTA and 0.2ng/µl Cy-3-conjugated 2'O-methyl sense oligonucleotide RNA probe; Integrated DNA Technologies) and coverslips were incubated at 80°C for 2 hours. Coverslips were washed three times for 30 minutes each in washing buffer (50% formamide, 0.5x SSC in distilled water). The hybridisation solution was then made up again but using the AlexaFluor488-conjugated antisense oligonucleotide probe (0.2ng/µl) and coverslips were incubated at 80°C for a further 2 hours, followed by three washes in washing buffer as before. Coverslips were washed for 10 minutes three times in 0.5x SSC and finally washed once with PBS. Samples were blocked for 30 minutes (10% donkey serum, 0.1% Triton-X in PBS) and stained with DAPI and Tuj1 according to the immunofluorescence protocol described above.

Manufacture of microfluidic devices

PDMS prepolymer and catalyst (10:1) were mixed thoroughly and poured into a master mould, and subsequently cured in an oven for 2h at 40 °C. Cured PDMS was removed from the master mould and 8 mm reservoirs were punched out. Irreversible bonding was achieved using a plasma cleaner.

Supplementary Figures

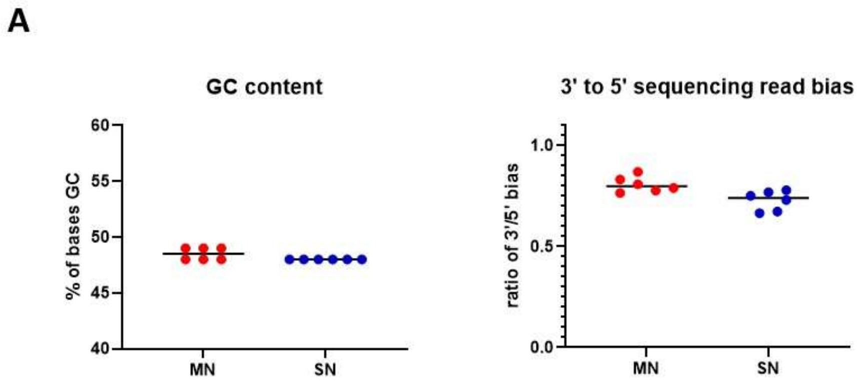

Supplementary Figure 1: Quality control of sequencing data generated in this study. GC content and 3’/5’ ratio of sequencing coverage shows small differences only.

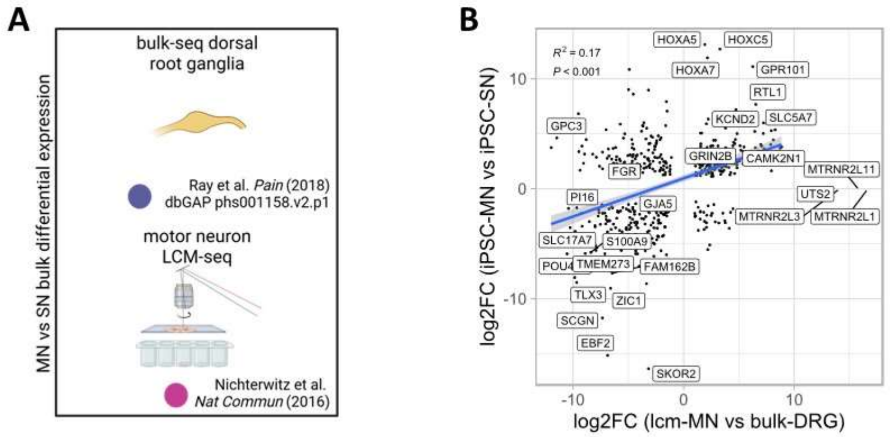

Supplementary Figure 2: Comparison of iPSC-MN vs SN differential expression with bulk datasets confirms findings from single cell data. A: Experimental design showing sources for bulk motor and sensory sequencing datasets used to determine differential expression between these two cell types. **B:** Scatter plot and linear correlation of differentially expressed genes between motor and sensory iPSC neurons versus differential expression between the bulk adult datasets, demonstrate a significant correlation between the two comparisons.

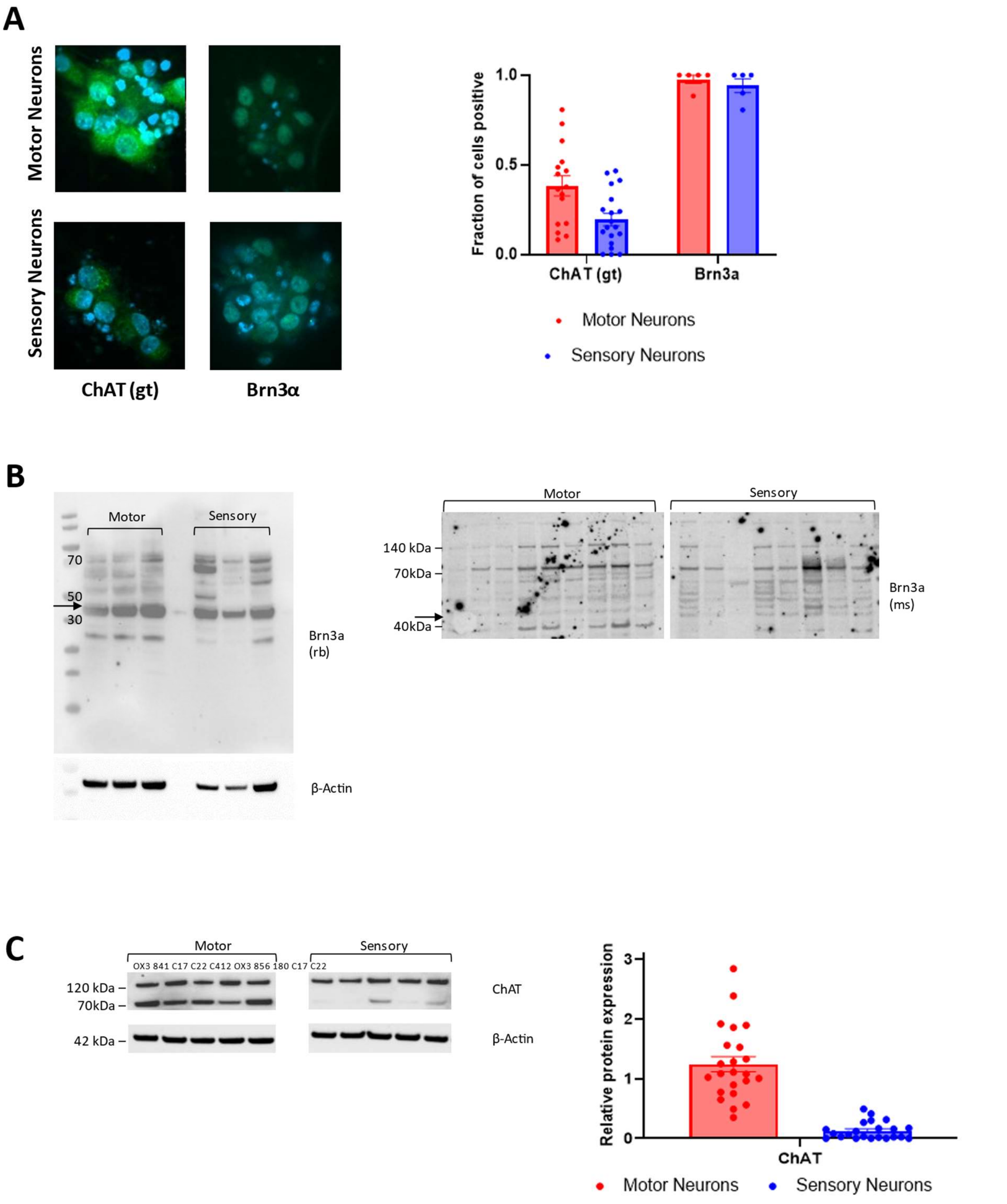

Supplementary Figure 3: Additional immunofluorescence and western blot analysis of motor and sensory neuron markers. A: Immunofluorescence analysis of Brn3a (rabbit) antibody showing ubiquitous staining in both iPS-MNs and iPS-SNs (n=1 differentiation), as well as additional ChAT staining (goat) staining with ChAT+ cells counted according to a fluorescence threshold confirming the presence of a higher proportion of ChAT+ cells in iPSC-MN cultures compared with iPSC-SN cultures ($p < 0.05$, t-test, n=2 differentiations). **B:** Example western blots for Brn3a rabbit (rb) and mouse (ms) antibodies showing a nonspecific staining pattern. **C:** Example western blot and quantification of ChAT staining

with ChAT (goat), which shows a specific band at the expected molecular weight and a nonspecific band at 120kDa. Quantification of the 70kDa band confirms enrichment of ChAT in motor neurons using this antibody ($p < 0.05$, t-test).

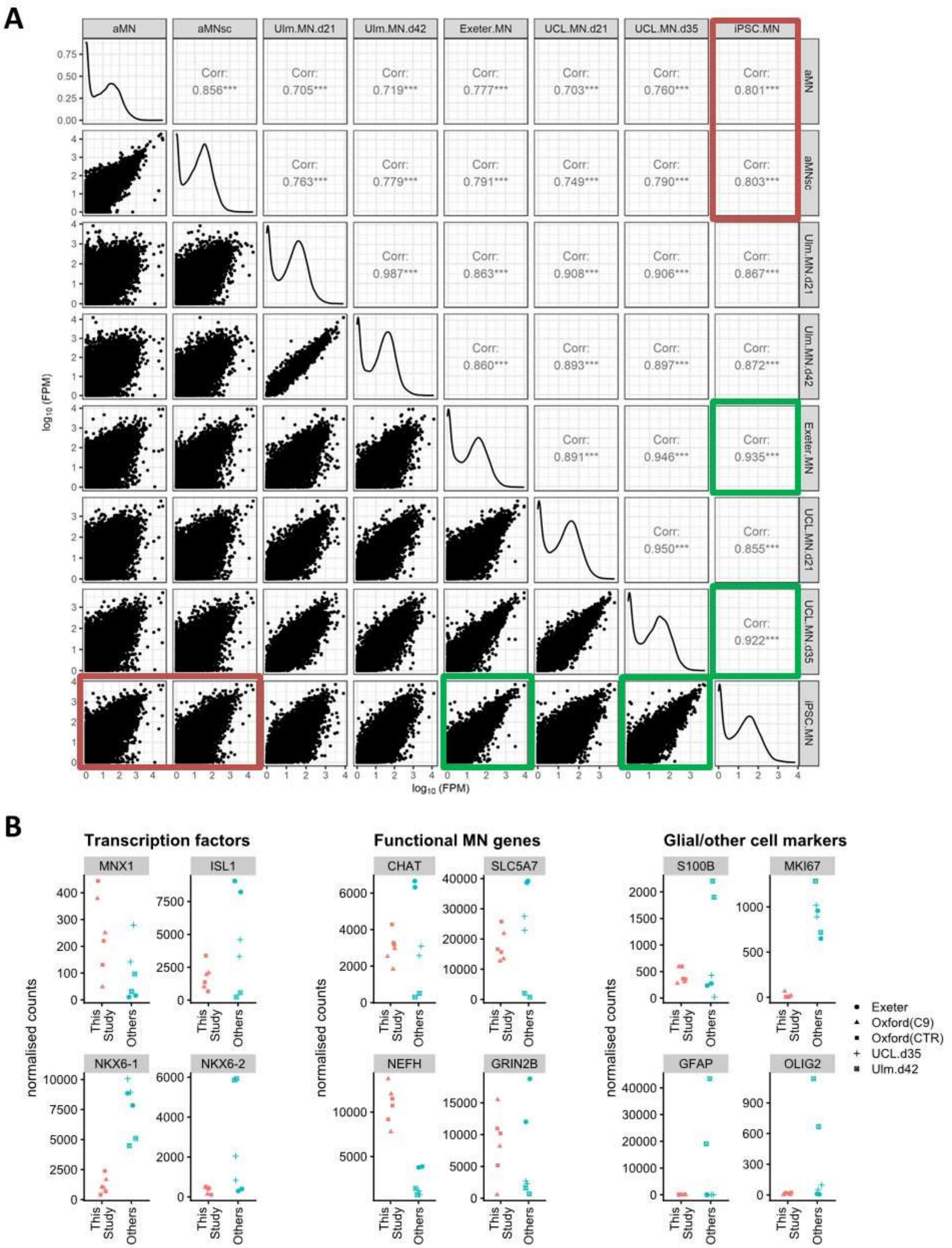

Supplementary Figure 4: Comparison of motor neuron differentiation in this study with previously published iPSC-MN datasets confirms similarity with other differentiations. A: High correlation between this study (labelled iPSC.MN) and two other mature motor neuron cultures (see green squares). Compared to other differentiations, the correlation with adult motor neurons derived from LCM-seq (aMN) and spinal cord single nucleus-seq (aMNsc) compared well with the other datasets (see red squares). Only control datasets were used

for this figure. **B.** Plot of normalised count from DESeq2 object shows similar levels of motor neuron transcription factors as well as expression of functional genes including ChAT, its transporter, neurofilament and ionotropic glutamate receptors. Our differentiation had low levels of non-neuronal cells and comparatively low levels of the proliferation marker *MKI67*. Exeter.MN = GSE203168, UCL.MN = GSE98288, Ulm.MN = GSE201407, aMN= GSE76514, aMNsc = GSE190442.

A

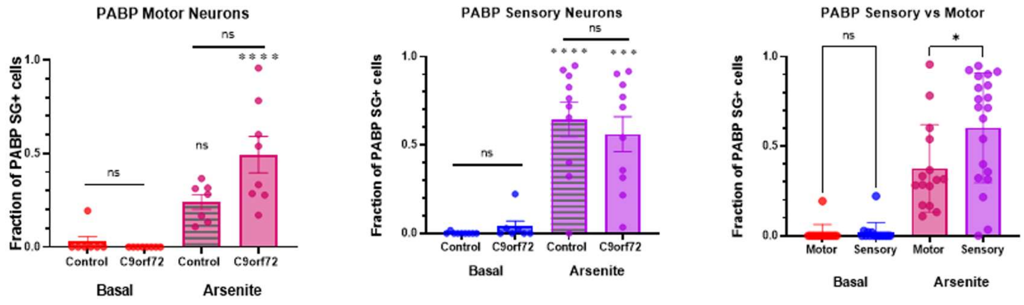

B

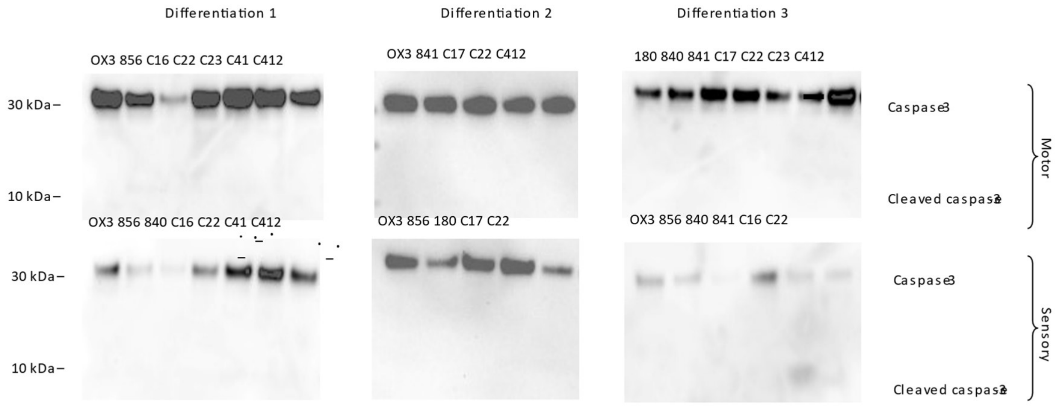

Supplementary Figure 5: Additional cellular phenotypes due to the *C9ORF72* HRE expansion in iPSC-MNs and iPSC-SNs. A: No difference in arsenite-induced PABP+ stress granule (SG) assembly between controls and *C9ORF72*+ iPSC-MNs or iPSC-SNs. Independent of the mutation, iPSC-SNs had a higher frequency of SGs following 1h Arsenite stress than iPSC-MNs. Stars denote significant differences between the basal stressed condition, n=2 differentiations. **B:** No staining of for cleaved caspase-3 (10kDa) when staining for full length caspase 3. n=3 differentiations.

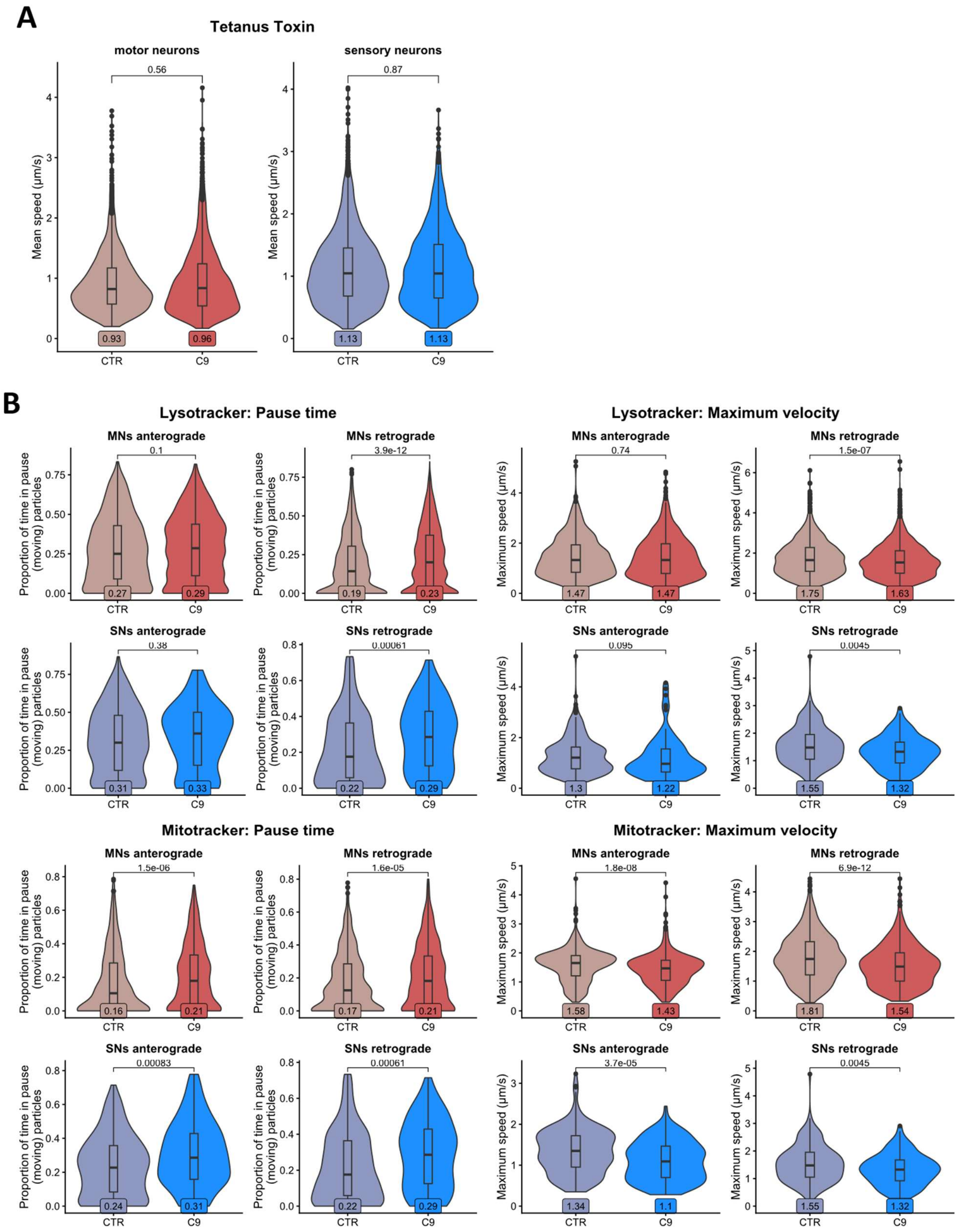

Supplementary Figure 6: Axonal transport of tetanus toxin and additional Lysotracker and Mitotracker data. A: No difference between controls and *C9ORF72*+ iPSC-MNs or iPSC-SNs in retrograde axonal transport of fluorescently labelled tetanus toxin. **B:** Increase in pause

times and decrease maximum velocity occur in lysotracker retrograde transport and Mitotracker bidirectional transport.
