# Supplementary material for: Cellular and axonal transport phenotypes due to the C9ORF72 HRE in iPSC motor and sensory neurons
Source: Stem Cell Reports. 2024 Jun 13;19(7):957–72. doi: 10.1016/j.stemcr.2024.05.008 (PMC11252479; doi:10.1016/j.stemcr.2024.05.008)
Supplement: Document S2. Article plus supplemental information [file mmc2.pdf]

Stem Cell Reports
Article
OPEN ACCESS

Cellular and axonal transport phenotypes due to the *C9ORF72* HRE in iPSC motor and sensory neurons

Jakub Scaber,[1,2,6,*] Iona Thomas-Wright,[1,2] Alex J. Clark,[1,3] Yinyan Xu,[1,2,4] Björn F. Vahsen,[1,2] Mireia Carcolé,[5] Ruxandra Dafinca,[1,2] Lucy Farrimond,[1,2] Adrian M. Isaacs,[5] David L. Bennett,[1] and Kevin Talbot[1,2,*]

[1]Nuffield Department of Clinical Neurosciences, University of Oxford, John Radcliffe Hospital, OX3 9DU Oxford, UK
[2]Kavli Institute for Nanoscience Discovery, University of Oxford, Dorothy Crowfoot Hodgkin Building, OX1 3QU Oxford, UK
[3]Centre for Neuroscience, Surgery and Trauma, Blizard Institute, Queen Mary University, E1 2AT London, UK
[4]Chinese Academy of Medical Sciences (CAMS), CAMS Oxford Institute (COI), Nuffield Department of Medicine, University of Oxford, OX3 7FZ Oxford, UK
[5]UK Dementia Research Institute at UCL and Department of Neurodegenerative Disease, UCL Queen Square Institute of Neurology, WCIN 3BG London, UK
[6]Lead contact
*Correspondence: jakub.scaber@ndcn.ox.ac.uk (J.S.), kevin.talbot@ndcn.ox.ac.uk (K.T.)
https://doi.org/10.1016/j.stemcr.2024.05.008

SUMMARY

Induced pluripotent stem cell (iPSC)-derived motor neurons (MNs) from patients with amyotrophic lateral sclerosis (ALS) and the *C9ORF72* hexanucleotide repeat expansion (HRE) have multiple cellular phenotypes, but which of these accurately reflect the biology underlying the cell-specific vulnerability of ALS is uncertain. We therefore compared phenotypes due to the *C9ORF72* HRE in MNs with sensory neurons (SNs), which are relatively spared in ALS. The iPSC models were able to partially reproduce the differential gene expression seen between adult SNs and MNs. We demonstrated that the typical hallmarks of *C9ORF72*-ALS, including RNA foci and dipeptide formation, as well as specific axonal transport defects, occurred equally in MNs and SNs, suggesting that these *in vitro* phenotypes are not sufficient to explain the cell-type selectivity of ALS in isolation.

INTRODUCTION

Amyotrophic lateral sclerosis (ALS) is a currently incurable neurodegenerative disease with a lifetime risk of 1:400 (Johnston et al., 2006). Its hallmark is the preferential degeneration of motor neurons (MNs) in the spinal cord and Betz cells in the corticospinal tract, with relative sparing of other cell types such as sensory neurons (SNs) (Kawamura et al., 1981), the visual system (Geser et al., 2008), the oculomotor nucleus (Okamoto et al., 1993), and the pelvic floor motor system (Kihira et al., 1997). The molecular basis for this anatomical selectivity remains obscure and may be due to cell autonomous effects in MNs or contributions from non-neuronal cell types to MN degeneration (Vahsen et al., 2021) or arise from the inherent vulnerability of the network architecture of the voluntary motor system (Talbot, 2014).

ALS is a clinically, pathologically, and genetically heterogeneous condition (Feldman et al., 2022), but the identification of multiple autosomal dominantly inherited variants of high penetrance has allowed the study of specific molecular subtypes. The commonest of these is a hexanucleotide expansion (HRE) in the first intron of *C9ORF72*, which accounts for up to 50% of all ALS cases with a family history of ALS or frontotemporal dementia. *C9ORF72* HRE-positive ALS causes typical TAR DNA-binding protein 43 (TDP-43) positive pathology but also results in a reduction in *C9ORF72* transcript levels, accumulation of repeat RNA in intracellular foci, and inclusions of dipeptide protein aberrantly translated from the hexanucleotide RNA. Induced pluripotent stem cell (iPSC) MNs have successfully recapitulated these *C9ORF72* HRE-dependent features with the qualification that dipeptide protein can be detected in cell lysates, but iPSC MNs do not have intracellular dipeptide inclusions (Braems et al., 2020). In addition, several disease-specific cellular phenotypes have been reported, including reduced survival, activation of autophagy markers (Braems et al., 2020), altered stress granule dynamics (Abo-Rady et al., 2020; Dafinca et al., 2016), impaired axonal transport (Abo-Rady et al., 2020; Fumagalli et al., 2021; Mehta et al., 2021), and nucleocytoplasmic transport (Zhang et al., 2015). However, key pathological hallmarks of ALS, mislocalization of TDP-43 or formation of cytoplasmic inclusions, have not been reliably demonstrated.

Thus far, modeling of ALS using iPSCs has predominantly focused on cell types thought to be selectively affected by ALS or frontotemporal dementia, such as MNs, cortical neurons, and glial cells (Giacomelli et al., 2022). A complementary approach, using iPSCs to differentiate cell types which are relatively spared in ALS, has the potential to provide insights into intrinsic vulnerability and resistance in disease. In ALS, SNs are an ideal candidate to study the specificity of iPSC MN phenotypes, as they traverse the same peripheral environment as spinal MNs and have similarly long axons. Although mild SN pathology is observed in ALS mouse models, it does not result in

This is an open access article under the CC BY license (http://creativecommons.org/licenses/by/4.0/).

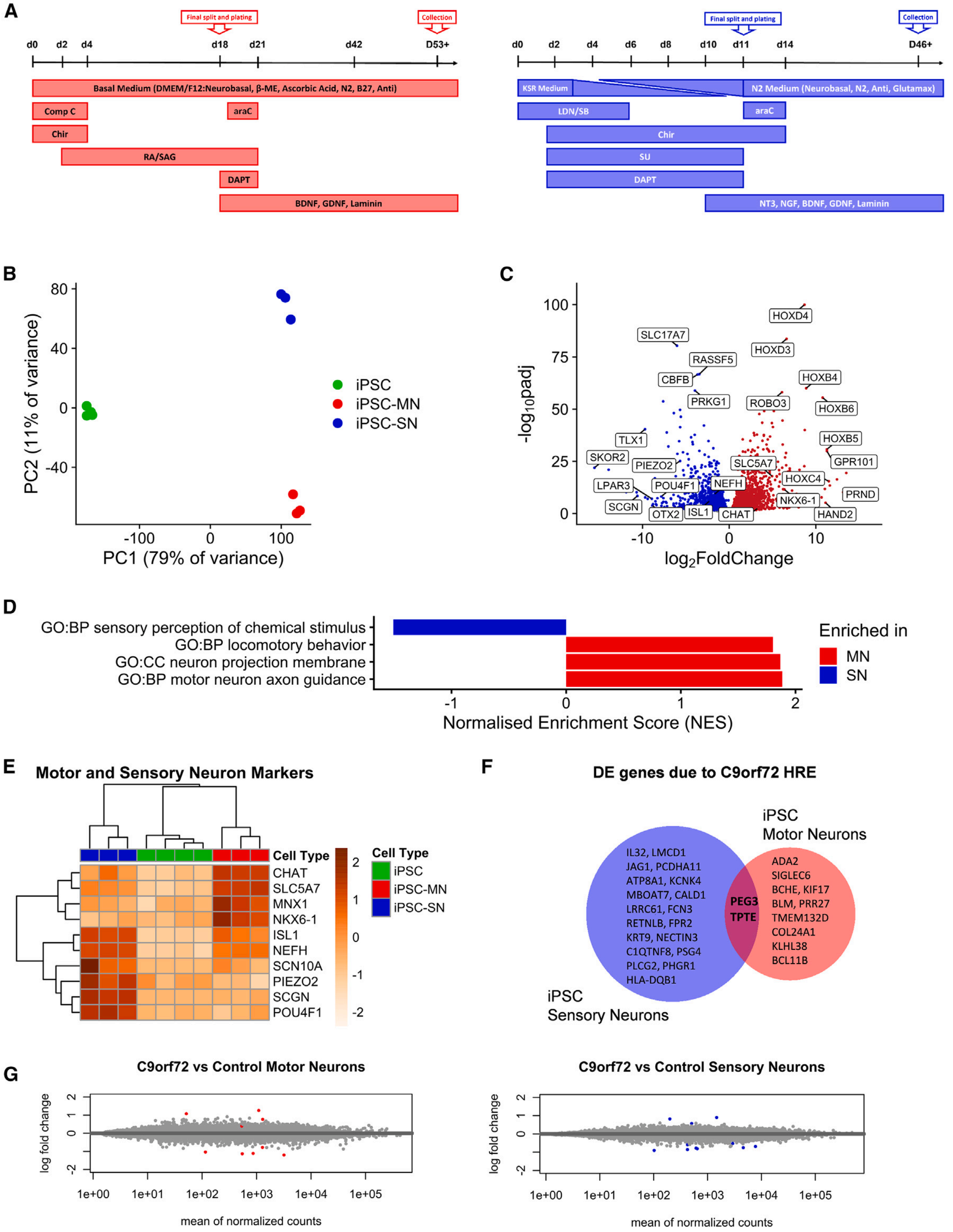

Figure 1. Sensory and motor neuron differentiations from iPSCs result in cell populations that recapitulate expected biological differences

(A) Schematic diagrams showing sensory and motor neuron differentiations. Anti, antibiotic-antimycotic; araC, cytosine arabinoside; BDNF, brain-derived neurotrophic factor; β-ME, β-mercaptoethanol; Chir, Chir 99021; comp C, compound C; GDNF, glial cell-derived neurotrophic factors; NT3, neurotrophin 3; NGF, nerve growth factor; RA, retinoic acid; SAG, smoothened agonist.

(legend continued on next page)

changes to cell numbers (Guo et al., 2009). Abnormal SN electrophysiology has been reported in human ALS patients, but the effects are largely sub-clinical in contrast to the debilitating motor symptoms caused by MN loss (Heads et al., 1991; Tao et al., 2018).

Here, we describe an in-depth phenotypic and molecular characterization of iPSC-derived SNs and MNs in parallel and investigate the effect of the *C9ORF72* HRE on both cell types, with the aim of elucidating factors that make MNs specifically vulnerable to degeneration.

RESULTS

iPSC-derived MNs and SNs have divergent transcriptomes that recapitulate their cell type

We generated bulk RNA sequencing data from iPSC MN and iPSC SN lines derived from three healthy control lines. Cells were harvested five weeks after final plating (day *in vitro* [DIV] 53 for iPSC MN and DIV 46 for iPSC SN (Figure 1A).

The first two principal components accounted for 90% of the total variance. As expected, the first principal component separated iPSCs from iPSC MNs and iPSC SNs and accounted for 79% of total variance. The second principal component separated iPSC MNs from iPSC SNs and accounted for 11% of the variance (Figure 1B). 5,906 protein-coding genes were found to be differentially expressed between iPSC MNs and iPSC SNs (Figure 1C), and gene set enrichment analysis (GSEA) confirmed appropriate enrichment of the relevant Gene Ontology categories for both cell types, after semantically filtering for terms relevant to the nervous system (adjusted $p < 0.05$, Figure 1D).

Subsequently, we used the RNA sequencing results to visualize marker genes that are used to identify MNs and SNs. The transcription factors *NKX6.1* and *MNX1* (encoding Hb9), as well as the cholinergic markers *CHAT* (encoding choline acetyltransferase) and *SLC5A7* (encoding the choline transporter), were expressed specifically in MNs. SN-specific markers were found to be the transcription factor *POU4F1* (encoding Brn3α), the mechanosensory receptor *PIEZO2*, and the calcium-binding protein *SCGN* (encoding secretagogin). *ISL1* and *NFH* were increased in both MNs and SNs compared to iPSCs. The use of the aforementioned markers resulted in the correct supervised clustering of iPSC MNs, iPSC SNs, and iPSCs (Figure 1E).

We then compared the aforementioned control cell lines with iPSC MN and iPSC SN lines from three different *C9ORF72* HRE carriers. In contrast to the extensive differential expression changes seen between iPSC MNs and iPSC SNs, only 12 genes were differentially expressed between *C9ORF72* HRE and control iPSC MNs and 21 genes between *C9ORF72* HRE and control iPSC SNs (Figure 1F), highlighting the subtle transcriptional consequences of the pathogenic variant in this model system even after prolonged growth in culture, an observation supported by a narrow $\log_2$-fold change distribution in both cell types (Figure 1G). There was a significant overlap in the differential expression due to the *C9ORF72* HRE between the two cell types (2/21, $p < 0.001$), indicating a convergence of the two datasets.

Transcriptomic comparison of iPSC neurons with human postmortem datasets reveals differences between the iPSC models and the corresponding adult cell types

iPSC-derived neurons reflect low embryonic age even after prolonged culture (Ho et al., 2021); thus we sought to investigate how well iPSC-derived neurons model the differential expression between adult human SNs and MNs. We compared single-nuclear RNA sequencing of dorsal root ganglia neuronal nuclei following neuronal nuclei antigen (NeuN) pull-down (Nguyen et al., 2021) with the annotated MN fraction of a recent spinal cord single-cell dataset (Yadav et al., 2023). Biological replicates were created by pooling all relevant single-cell expression data for each tissue donor to create "pseudobulk" samples.

The heatmap of the transformed sample distance matrix demonstrated clustering of the iPSC-derived samples away from the postmortem samples but with appropriate separation of cell types within these clusters (Figure 2B). Supervised hierarchical clustering of known SN and MN marker genes on the other hand yielded the expected clustering by cell type, indicating the presence of common cell type-specific features between iPSC-derived neurons and their adult counterparts (Figure 2C). Compared with adult

(B) Principal-component (PC) analysis of iPSC MNs, iPSC SNs, and iPSCs.

(C) Volcano plot showing differentially expressed genes between iPSC MNs and iPSC SNs, with genes enriched in motor neurons in red (FDR <0.05) and those enriched in iPSC SNs in blue (FDR <0.05).

(D) Significant (FDR <0.05) gene ontology enrichment in iPSC MNs and iPSC SNs showing top enriched categories after filtering for neuronally relevant terms ("senso," "nerve," "neuro," "nervous," "spinal," "motor").

(E) Heatmap of mean normalized expression of known motor and sensory neuronal marker genes in iPSC MNs, iPSC SNs, and iPSCs. Marker genes correctly cluster into three categories: present in iPSC MNs only, present in iPSC SNs only, and present in both.

(F) Venn diagram of differentially expressed genes due to the *C9ORF72* HRE in iPSC MNs and iPSC SNs.

(G) MA plot of differential expression analysis between *C9ORF72*+ iPSC MNs and controls (red, FDR <0.05) and *C9ORF72*+ iPSC SNs and controls (blue, FDR <0.05).

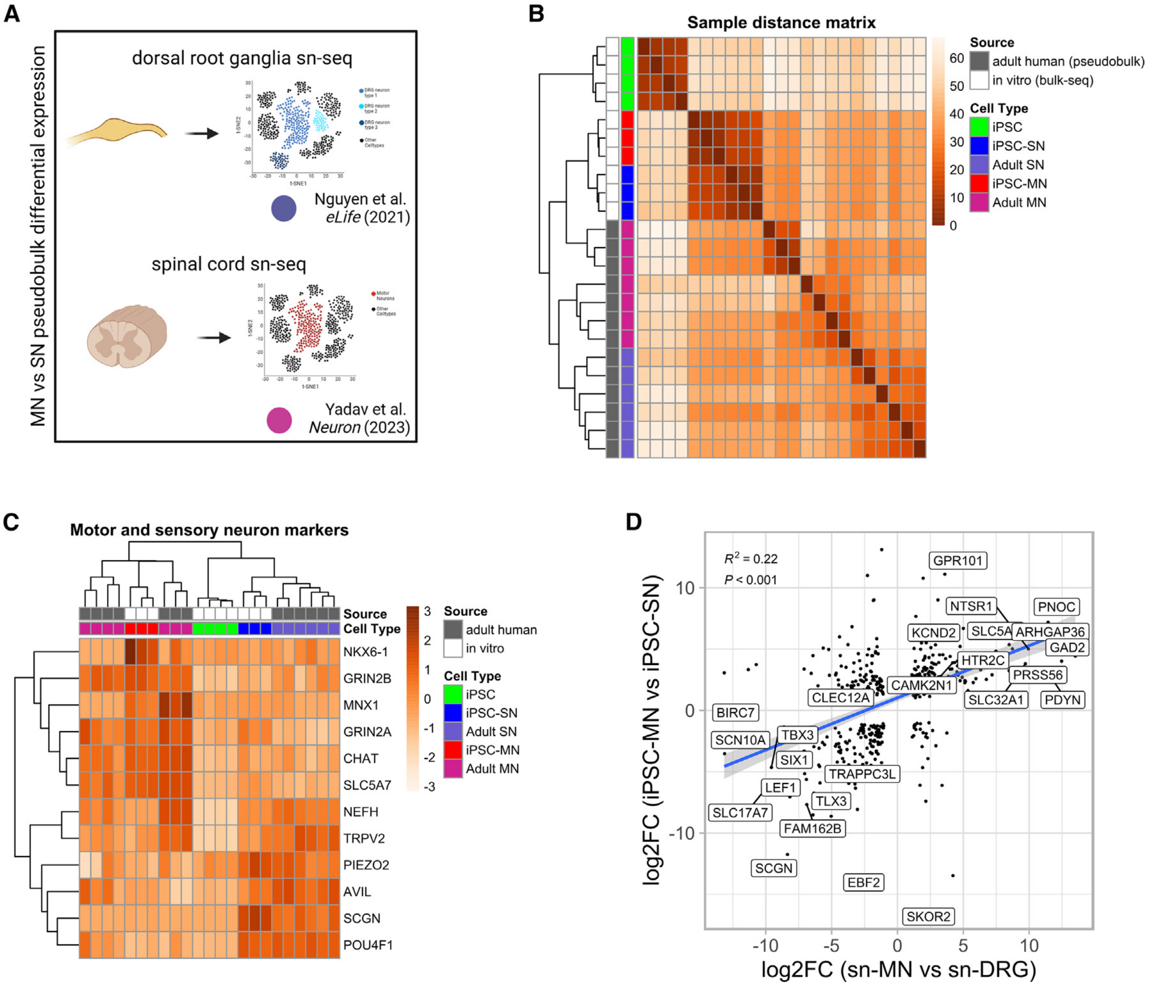

Figure 2. Comparison with single-cell adult datasets confirms relevance of iPSC model for studying cell-selective neuronal phenotypes but also reveals important differences

(A) Experimental design showing sources for motor and sensory single-nucleus sequencing datasets used for pseudobulk differential expression.

(B) Sample distance matrix that includes all datasets used in this study demonstrates hierarchical clustering of *in vitro* samples away from postmortem datasets but correct separation of motor and sensory neurons within each cluster.

(C) Supervised clustering using heatmap of marker genes shows correct clustering of samples by cell type, independent of sample origin. The figure shows that some MN markers are consistent between iPSC MNs and postmortem samples but that there are also important differences between the model and adult tissue.

(D) Scatterplot and linear correlation of differentially expressed genes between motor and sensory iPSC neurons versus differential expression between the pseudobulk adult datasets, demonstrate a significant correlation between the two comparisons.

MNs, iPSC MNs had similar expression of *CHAT*, *SLC5A7*, and N-methyl-D-aspartate (NMDA) receptor genes, but lower expression of *NEFH*, instead expressing developmental ventral neuronal cord transcription factor *NKX6.1*.

To specifically examine the utility of the iPSC model for the study of selective vulnerability, we assessed whether genes differentially expressed between motor and sensory iPSC-derived neurons were also differentially expressed in the postmortem dataset. For this, we plotted differentially expressed genes (false discovery rate [FDR] <0.05) above an absolute log_2-fold threshold of 1 and found a significant correlation between the two comparisons (R^2 = 0.22, Figure 2D). As the validity of MN clusters in single-cell spinal cord datasets is debated (Gautier et al., 2023), we performed the same analysis against a second adult dataset, created by comparing available laser capture microdissection RNA sequencing (LCM-seq) of pooled samples containing 120 captured MN nuclei from each donor (Nichterwitz et al., 2016), with an available RNA sequencing dataset of whole dorsal root ganglia (Ray

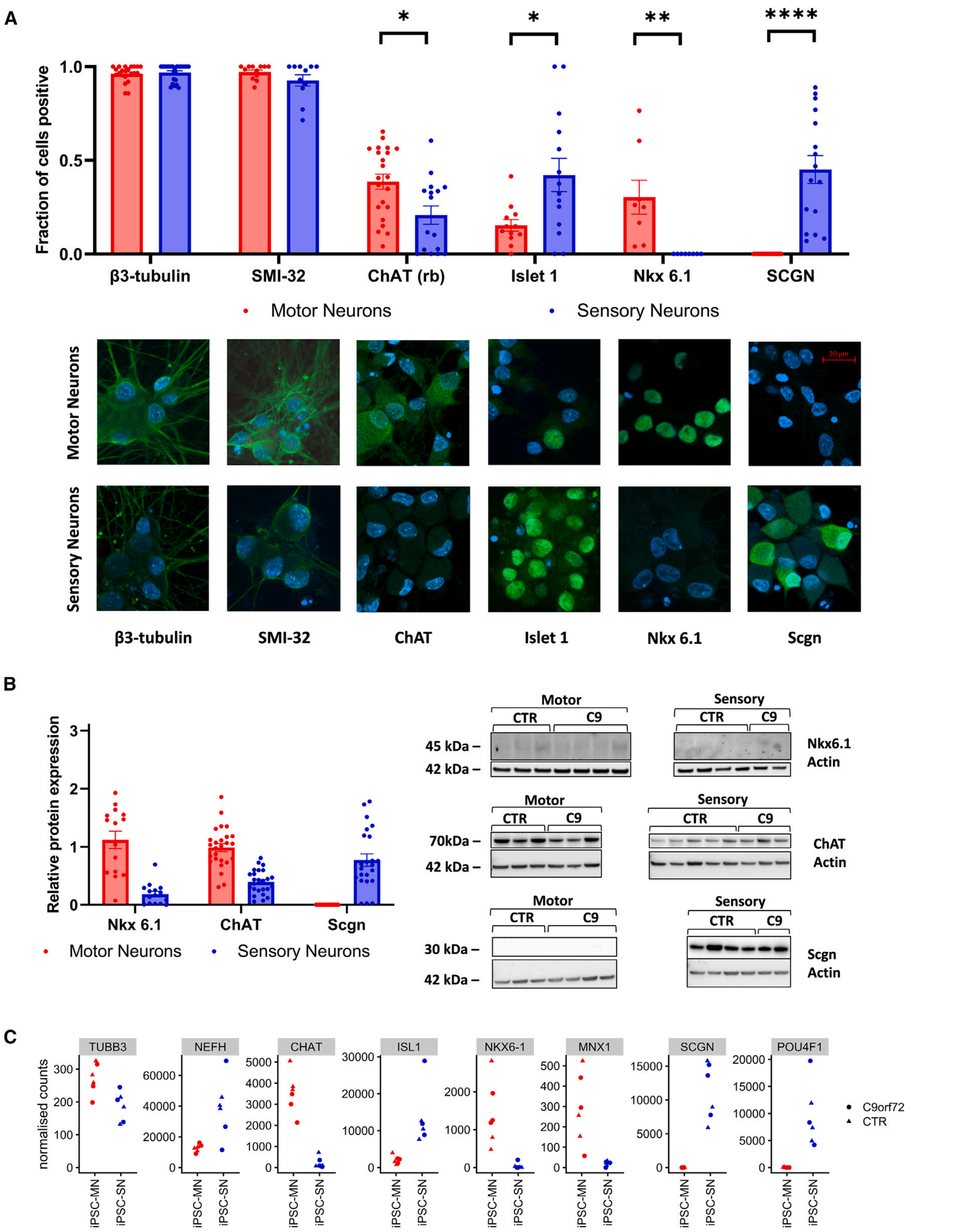

Figure 3. iPSC MNs and iPSC SNs can be distinguished by immunofluorescence and western blotting

(A) Immunofluorescence imaging of iPSC MN and iPSC SN cultures five weeks after final plating, with representative images of the marker in each cell type below the bar graph. High proportion of β-3 tubulin- and SMI-32-positive cells confirms high efficiency and no difference in

(legend continued on next page)

et al., 2018), which yielded similar results (R^2 = 0.17, Figure S2).

Only a subset of antibody markers used to specify MNs and SNs can distinguish these cell types when derived from iPSCs

Despite specific expression of MN and SN markers linking iPSC neurons to their adult cell type, using immunofluorescence, we found that only two antibodies reproducibly distinguished iPSC MNs or iPSC SNs: Nkx6.1 was present only in iPSC MNs, and secretagogin, which is known to label the calcitonin gene-related peptide (CGRP) subtype of SNs, was solely present in iPSC SNs (Figure 3A). Unfortunately, we were not able to find an antibody for the pan-sensory neuronal marker Brn3α, as tested antibodies stained both iPSC MN and iPSC SN nuclei (Figures S3A and S3B).

The MN marker choline acetyltransferase (ChAT) is known to be expressed in some dorsal root ganglia cells and has diffuse cytoplasmic staining (rabbit monoclonal ChAT in Figure 3A; goat ChAT in Figure S3C). We used a fixed-threshold method, which demonstrated a difference in ChAT positivity between the two cell types. Finally, we were able to show that both iPSC MNs and iPSC SNs were positive for β3-tubulin and SMI-32, a heavy-chain neurofilament marker, indicating high neuronal purity (near 100%) following antimitotic treatment. As expected, islet1 was present in both cell types, but at a higher frequency in iPSC SNs, consistent with its differential gene expression in our cultures ($p < 0.05$, Figure 3A).

Western blot analysis on whole-culture lysates confirmed the immunofluorescence findings, with Nkx6.1 and secretagogin present in the relevant cell types, and ChAT being more abundant in iPSC MNs ($p < 0.05$, Figures 3B and S3C).

RNA sequencing data confirmed the presence of selective expression of the aforementioned genes, regardless of the *C9ORF72* HRE genotype (Figure 3C). Given the lack of a single standardized MN differentiation protocol, we also compared the gene expression profile of iPSC MNs in this study against three other published datasets of control iPSC MNs (Hall et al., 2017; Hawkins et al., 2022; Sommer et al., 2022), including one that reported >60% islet1-positive neurons. There was a high correlation with these datasets and with postmortem datasets (Figure S4A). Expression of MN markers was similar to that in other iPSC MN studies. Compared to all remaining studies, the iPSC MNs in this study had a particularly low expression of the proliferation marker *MKI67* (Figure S4B).

MNs and SNs show similar expression of RNA foci and dipeptides

Fluorescence *in situ* hybridization was performed for both sense and antisense RNA foci, which were found at similar frequencies in both iPSC SNs and iPSC MNs in *C9ORF72* HRE-positive lines (Figure 4A). No differences in C9orf72 protein or *C9ORF72* mRNA expression were seen in mutant iPSC MNs or iPSC SNs compared to controls, though a small difference would not have been captured by this study given its sample size (Figure 4B). An ELISA for poly-GP and poly-GA confirmed the presence of these dipeptides in both mutant iPSC SNs and iPSC MNs at similar levels (Figure 4C).

iPSC MNs have fewer stress granules and a higher cytoplasmic/nuclear ratio of TDP-43 compared to iPSC SNs

To ascertain if reported iPSC MN phenotypes due to the *C9ORF72* HRE can be found in iPSC SNs, we investigated stress granule formation, TDP-43 distribution, survival, and expression of proapoptotic markers.

Ras-GTPase-activating protein binding protein (G3BP)-positive stress granules were rarely present under basal conditions but were highly abundant in response to arsenite stress, with no differences between control and *C9ORF72* HRE-positive cell lines in either iPSC MNs or iPSC SNs. However, iPSC SNs had more abundant arsenite-induced stress granules compared to iPSC MNs (Figure 5A). Results were independently replicated using poly(A)-binding protein (PABP) as an alternative marker (Figure S5A).

No TDP-43 puncta were observed in any cell lines, and there were no differences in nuclear/cytoplasmic staining intensity of TDP-43 between control and mutant cell lines in iPSC MNs and iPSC SNs under basal or stress conditions, though iPSC MNs had consistently higher relative cytoplasmic TDP-43 localization compared to iPSC SNs, under all conditions (Figure 5B).

Increased apoptosis and reduced survival have previously been reported in *C9ORF72* HRE-positive iPSC MNs (Abo-Rady et al., 2020; Shi et al., 2018). We performed longitudinal cell viability and cell death assays using the RealTime

neurogenesis between both cultures. Nkx 6.1, only found iPSC MNs ($p < 0.01$), and secretagogin (Scgn), only found in iPSC SNs ($p < 0.0001$) confirm functional divergence of the two cultures. After setting of a threshold, higher levels of ChAT+ cells are seen in iPSC MNs compared to iPSC SNs ($p < 0.05$) and islet1+ cells were more frequent in iPSC SNs ($p < 0.05$). $n = 2$ differentiations.

(B) Western blotting confirmed correct molecular weight and cell-type specificity of the Nkx 6.1 ($p < 0.01$) and secretagogin antibodies ($p < 0.01$), as well as the relative cell-type specificity of the ChAT antibody at the expected molecular weight of 70kDa ($p < 0.05$). $n = 3$ differentiations.

(C) Gene expression of key motor and sensory neuron marker genes from RNA Sequencing data (derived from DESeq2 normalised counts matrix) confirms cell-type specificity and similar expression levels in *C9ORF72*+ and control neurons.

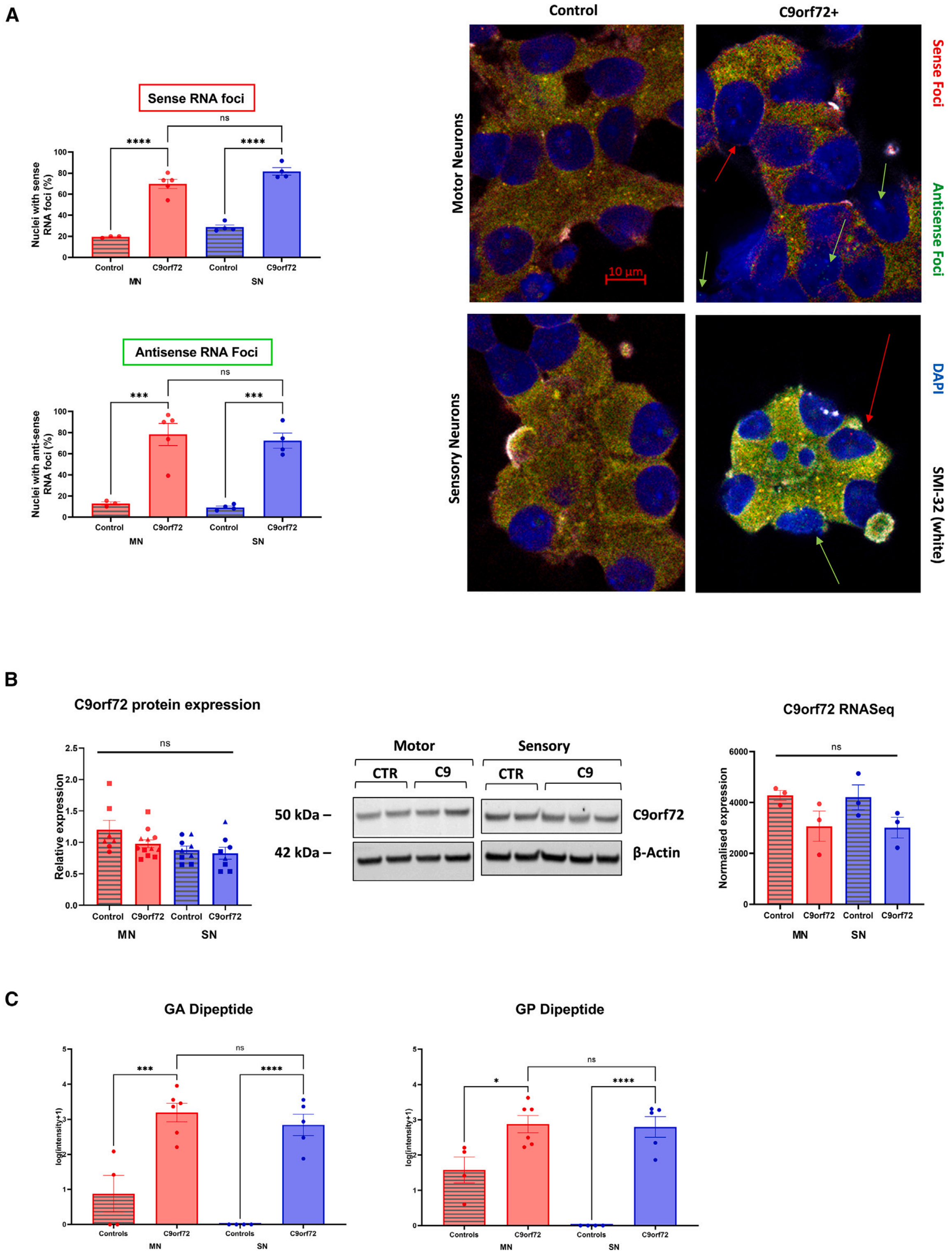

Figure 4. iPSC MNs and iPSC SNs from *C9ORF72*-positive patients have similar levels of RNA foci and dipeptide protein

(A) RNA sense ($p < 0.0001$) and antisense ($p < 0.01$) foci were detected in both *C9ORF72*+ iPSC MNs and iPSC SNs, with detection of nonspecific background staining only in controls. No significant differences in the abundance of RNA foci were seen between the two cell types ($n = 1$ differentiation).

(legend continued on next page)

Glo and CellTox assays following treatment with 0.5 mM sodium arsenite for 1 h, and this did not reveal a difference between *C9ORF72* HRE-positive cell lines and controls in either iPSC MNs or iPSC SNs at two different culture time points (Figure 5C). Similarly, we could not detect cleaved caspase 3 or changes in the relative expression of the proapoptotic proteins Bax and Puma in control or *C9ORF72* HRE-positive iPSC MNs and SNs even after exposure to arsenite (Figures S5B and S5D).

C9ORF72 HRE results in specific slowing of retrograde lysosomal transport and bidirectional slowing of mitochondrial transport in both MNs and SNs

As the axon is the distinguishing feature of neurons and could be a site of early neuronal pathology in ALS, based on evidence from genetic studies (Zhang et al., 2022), animal models (Sleigh et al., 2020; Williamson and Cleveland, 1999), and previous reports in *C9ORF72* HRE-positive iPSC MNs (Dafinca et al., 2020; Mehta et al., 2021), we sought to assess multiple modalities of transport using custom-made microfluidic devices. Samples were assessed at 21 days following the final seeding of neurons. We used LysoTracker to assess both anterograde and retrograde lysosomal transport and fluorescently labeled tetanus toxin (HcT), which preferentially labels retrograde endosomal transport. Finally, we also used MitoTracker to assess mitochondrial transport. Samples were analyzed using Difference Tracker, and image processing and analysis were fully automated (Figure 6A).

A total of 72,959 particles were identified, of which 33,551 passed quality control (non-stationary; all particles moving horizontally in the video). A total of 15,639 LysoTracker particles, 12,609 tetanus toxin particles, and 5,303 MitoTracker particles were analyzed. A reduction in the mean velocity of travel was observed in lysosomal transport in both iPSC MNs and SNs with the *C9ORF72* HRE compared to controls ($p < 1e{-}06$), and this was specific for retrograde but not anterograde transport in both cell types (Figure 6B). Retrograde transport of tetanus toxin was not affected in either cell type (Figure S6A). Using MitoTracker, we observed a slowing of mitochondrial transport due to the *C9ORF72* HRE by approximately 15% in both iPSC MN and SNs with the *C9ORF72* HRE ($p < 0.001$), and this was seen in both anterograde and retrograde direction in both cell types.

More detailed analysis of the characteristics of travel did not point to a specific mechanism for the axonal slowing observed in this study, as it was accompanied by both an increased proportion of time of particles spent in pause and slowing of maximal velocity in all relevant MitoTracker and LysoTracker experiments (Figure S6B).

DISCUSSION

In this study, we carried out a detailed phenotypic characterization of iPSC MNs and SNs from patients carrying the *C9ORF72* HRE, with the aim of finding MN-specific phenotypes that could give rise to selective vulnerability. We provide evidence for similar levels of RNA sense and antisense foci as well as GA and GP dipeptide production in iPSC MNs and SNs. Both RNA foci and dipeptide have been postulated to be potential gain-of-function mechanisms by which the *C9ORF72* HRE could disturb cellular homeostasis, eventually resulting in ALS. In this study we were not able to replicate differences in stress granule formation and TDP-43 distribution or survival after arsenite stress due to the *C9ORF72* HRE. We then demonstrate that deficits in retrograde lysosomal and bidirectional mitochondrial transport in iPSC-derived neurons from ALS patients with the *C9ORF72* HRE are not MN specific but that matching deficits can also be observed in iPSC SNs from the same patients. Given that SNs are relatively spared in ALS, this suggests that changes in axonal transport due to the *C9ORF72* HRE observed here and in previous studies are unlikely to be responsible for selective vulnerability in MNs in isolation. A contribution of *C9ORF72* HRE-dependent slowing of axonal transport to the disease in the context of an aging nervous system with its complex cellular connections and in combination with other pathological processes cannot be excluded.

The mechanism of how the *C9ORF72* HRE causes axonal transport abnormalities remains under investigation. The specificity of deficits only in retrograde lysosomal transport and bidirectional mitochondrial transport, with sparing of endosomal transport and lysosomal anterograde transport, suggests that axonal degeneration through general failure of microtubule homeostasis is unlikely. One *in vitro* study suggested that the presence of arginine-containing dipeptides causes the mitochondrial transport machinery to stall (Fumagalli et al., 2021). Another study argued that abnormal mitochondrial bioenergetics may result in the slowing of mitochondrial transport (Mehta et al., 2021), although we could not replicate the differential expression of mitochondrially encoded genes reported in that study.

(B) No significant differences in C9orf72 protein and *C9ORF72* mRNA expression in *C9ORF72*+ MNs or SNs ($n = 3$ differentiations, marked by different symbols).
(C) ELISA quantification of GA and GP dipeptide protein in motor and sensory neurons. Analysis of pooled protein from up to 3 differentiations, 1 dot = 1 cell line.

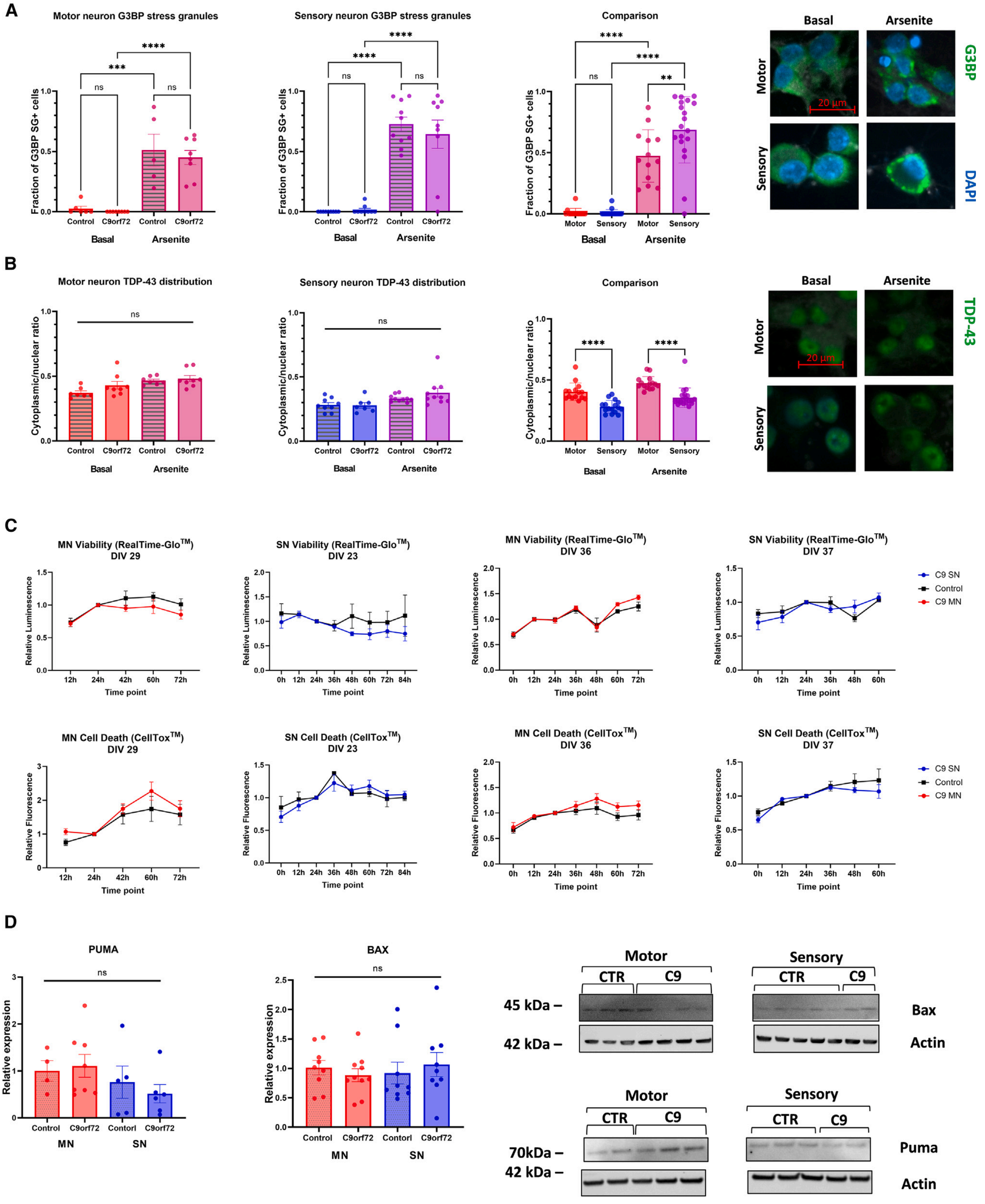

(legend on next page)

The very low number of differentially expressed genes in our study indicates that both the mutation and its axonal phenotype are relatively well tolerated in the developing MN and that the axonal transport deficits are not linked to the pathogenic variant through changes in transcriptional regulation.

In this study, we were not able to replicate abnormalities of cellular homeostasis that have been reported in studies of *C9ORF72* HRE iPSC MNs previously (Braems et al., 2020). Of note, a number of these findings were reported by our research group previously (Dafinca et al., 2016, 2020). Compared to previous studies, this study is characterized by longer time in culture, enabled by changes in the adhesion layer and the addition of an antimitotic treatment, which successfully removed contaminating non-neuronal dividing cells and reduced overall variance. We have not used stressors other than sodium arsenite in this study, and it is possible that prolonged or different stress conditions could result in a loss of cellular homeostasis due to the *C9ORF72* HRE in the lines presented here.

The absence of widespread metabolic disturbances is supported by lack of differential expression due to the *C9ORF72* HRE, independently replicated in both iPSC MNs and iPSC SNs. While our sample size is small, it argues against substantial metabolic disturbance in C9orf72 at basal conditions, in line with a recent large-scale transcriptomic study which compared over 300 patient-derived iPSC-derived MN lines with controls, which included 29 lines from *C9ORF72* HRE carriers, and did not find significant transcriptional derangements (Workman et al., 2023). In contrast to this prospective study, a meta-analysis of published smaller sequencing studies has found an apoptotic signal (Ziff et al., 2023), which may potentially indicate the presence of publication bias toward stronger phenotypes in existing smaller studies.

The presence of the *C9ORF72* HRE mutation is not a sufficient condition for the development of ALS, which occurs in adult MNs in the context of aging and a complex multicellular nervous system. This study demonstrates that iPSC-derived MNs and SNs have divergent identities and recapitulate some of the features of the adult cell type but also emphasizes differences. These may partly be explained by important limitations of the adult datasets used in this study, including the nuclear origin of the RNA in single-cell studies, the high glial component in both the single-cell and the laser-captured MNs, and the variability of postmortem datasets. Notwithstanding these inconsistencies, iPSC MNs remain transcriptomically distinct from their adult counterparts and may therefore potentially lack the characteristics required for ALS to manifest.

The study has two implications for the understanding of ALS modeling using iPSCs. Firstly, differential expression changes due to the ALS-causing *C9ORF72* HRE variant in iPSC-derived MNs are small compared to the differences between iPSC MNs and healthy adult MNs, and therefore any transcriptomic changes in the model may not be simply translatable to postmortem studies. Secondly, at least some *in vitro* phenotypes seen in iPSC-derived MNs with the *C9ORF72* HRE may be equally present in cell types that do not degenerate in ALS and therefore less likely to be primary drivers of cell-specific neurodegeneration.

The findings in this study raise important considerations for future investigations of the *C9ORF72* HRE. Understanding how to further encourage maturation of iPSC MNs toward the adult MN type will be crucial in neurodegenerative disease and may necessitate increasing the complexity of the model through functional stimulation or the addition of other cell types, which will require stringent control of experimental variability. The lack of a strong transcriptomic disease signature in *C9ORF72* HRE iPSC neurons is an important challenge in this context and will require further detailed study of different stress paradigms, including chronic stress and stress recovery, and may require more detailed methods than whole-cell quantification of mRNA and protein to capture subtle differences of cellular homeostasis.

In conclusion, this study demonstrates how the ability of iPSCs to generate divergent cell types can be harnessed to better understand cell-type specificity in ALS. It provides evidence that the axonal phenotypes reported in iPSC MNs are also present in iPSC SNs and therefore are unlikely to be the sole driver of selective vulnerability. Future work,

Figure 5. Lack of cellular phenotypes due to the *C9ORF72* HRE in iPSC SNs or iPSC MNs

(A) No difference in arsenite-induced G3BP + stress granule (SG) assembly between controls and *C9ORF72*+ iPSC MNs or iPSC SNs. Independent of the mutation, iPSC SNs had a higher frequency of SGs following 1 h Arsenite stress than iPSC MNs. Stars denote significant differences between the basal and stressed conditions, $n = 2$ differentiations.

(B) No difference between controls and *C9ORF72*+ iPSC MNs or iPSC SNs in either the basal condition or following 1 h of arsenite stress. Independent of the mutation, iPSC MNs had a higher ratio of cytoplasmic/nuclear TDP-43 staining in both the basal condition and after 1 h of arsenite stress.

(C) No differences in viability (Real-Time Glo) or cell death (CellTox) between controls and *C9ORF72*+ iPSC MNs or iPSC SNs at both an early and late time point ($n = 1$ differentiations).

(D) Quantification and representative images of staining for the proapoptotic markers PUMA and BAX, which did not show a difference between controls and *C9ORF72*+ iPSC MNs or iPSC SNs.

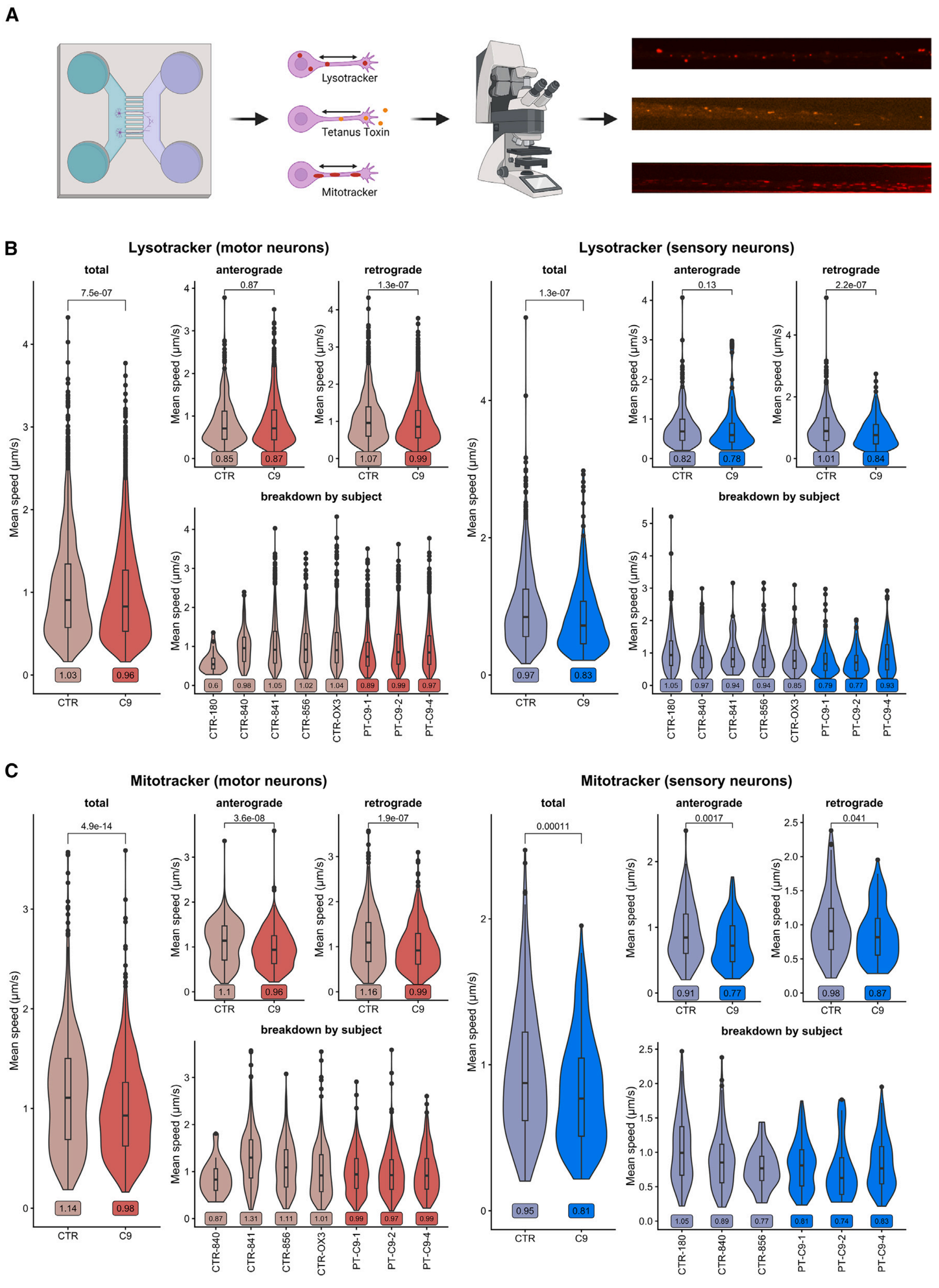

(legend on next page)

both in terms of developing the iPSC model systems and understanding the differences between adult MNs and SNs, will be required to understand the basis for selective vulnerability in ALS.

EXPERIMENTAL PROCEDURES

Resource availability

Lead contact

Further information and requests for resources and reagents should be directed to and will be fulfilled by the lead contact, Dr. Jakub Scaber (Jakub.scaber@ndcn.ox.ac.uk).

Materials availability

This study did not generate new unique reagents.

Data and code availability

The data generated in this study have been uploaded to the Gene Expression Omnibus repository GSE262929. The code used for the generation of RNA sequencing figures and for the imaging analysis is available at https://github.com/jscaber/sensorimotor.

Use of human-derived iPSCs in this study

Previously published iPSC lines from three patients (two clones from each patient), as well as five sex- and age-matched controls were used (Ababneh et al., 2020; Dafinca et al., 2016, 2020) and are summarized in Table S1. iPSC lines were derived from human skin biopsy fibroblasts collected under ethical approval granted by the South Wales Research Ethics Committee (WA/12/0186) and the South-Central Berkshire Research Ethics Committee (REC10/H0505/71) in the James Martin Stem Cell Facility, University of Oxford, under standardized protocols. Cells were passaged using EDTA (0.5 mM), expanded to produce consistent, frozen cell stocks for the study, and tested negative for mycoplasma using the MycoAlert mycoplasma detection kit (Lonza).

MN and SN differentiation

MN differentiation (Ababneh et al., 2020) and SN differentiation (Clark et al., 2021) followed previously published protocols with minor modifications, most notably including the addition of cytosine arabinoside in the MN differentiation and coating with polyethyleneimine and Geltrex at the final plating for both differentiations. The day of induction was defined as DIV 0. Detailed methods are available in the , and a schematic representation can be found in Figure 1A. Cells were harvested five weeks after final plating, which was DIV 45 for iPSC SNs and DIV 52 for iPSC MNs for all experiments except where specified. Growth factors used can be found in Table S4.

Protein extraction, quantification, and western blotting

Protein extraction, quantification using the bicinchoninic assay, and western blotting were carried out using standard laboratory practices. A total of three differentiations were performed. Across blots, data were normalized against the mean of MN controls on each blot. Detailed methods including antibodies used are available in the supplemental information.

Immunocytochemistry and fluorescence *in situ* hybridization

Cells were grown on glass coverslips and fixed five weeks after plating. Cells conditions were treated with 0.5 mM sodium arsenite 1 h prior to fixation and staining in relevant experiments. Immunocytochemistry was carried out using standard laboratory practices. Fluorescence *in situ* hybridization was carried according to previously published methods (Ababneh et al., 2020). Detailed methods including antibodies used are available in the supplemental information.

Live imaging of axonal transport using microfluidic devices

Microfluidic devices were produced according to a previously described protocol (Park et al., 2006) with detailed methods in the supplemental information. Channels were coated with Geltrex for at least 1 h before plating of ~25,000 neurons. During the duration of culture in the devices, a gradient was maintained with 50 μL additional medium and a 5-fold concentration of growth factors in the axonal wells compared to the cell body side.

Neurons were grown in microfluidic devices for 3 weeks after final seeding (DIV 32 for iPSC SNs, DIV 39 for iPSC MNs), as longer cultures resulted in degradation of the axons. Fluorescent molecular dyes were added to the medium 45 min prior to imaging. Separate axonal chambers were used for each dye, and chambers were never reused. LysoTracker Red DND-99 and MitoTracker Red CMXRos at concentrations of 70 nM were applied to both sides.

Figure 6. Specific slowing of endosomal transport and mitochondrial transport due to the *C9ORF72* HRE is seen equally in iPSC MNs and iPSC SNs

(A) Schematic showing a typical microfluidic chamber and the experimental setup with three fluorophores for imaging lysosomal, retrograde endosomal, and mitochondrial transport, including example still images from the respective movies.

(B) Results from LysoTracker analysis in iPSC MNs (red) and iPSC SNs (blue) showing slowing of lysosomal transport in both *C9ORF72*+ iPSC MNs and *C9ORF72*+ iPSC SNs compared to controls, which is seen only in retrograde and not anterograde transport. Lower panel shows breakdown between lines to give indication of biological variability. Violin plots with boxplot insert showing median, with mean indicated in the label below. *n* = 3 differentiations.

(C) Results from MitoTracker analysis in iPSC MNs (red) and iPSC SNs (blue) showing slowing of mitochondrial transport in both *C9ORF72*+ iPSC MNs and *C9ORF72*+ iPSC SNs compared to controls, which is seen in both retrograde and anterograde transport. Lower panel shows breakdown between lines to give indication of biological variability. Violin plots with boxplot insert showing median, with mean indicated in the label below. *n* = 3 differentiations.

Tetanus toxin was added to the axonal side only at a concentration of 40 nM. Microfluidic chambers were imaged in a Zeiss scanning disc confocal microscope with an incubation chamber containing 5% CO_2 and heated to 37°C. Imaging was performed using a 63x apochromat lens, on a single plane (to minimize bleaching and delay) with an image of the distal axonal groove taken every 2 s for a total of 10 min per experiment.

Live image analysis

Live image analysis was performed using the software package Difference Tracker (Andrews et al., 2010), which was automated using a custom Python script in Fiji (Schindelin et al., 2012). The raw output from the software was extracted and processed in R using a custom script, which added features such as velocity/direction and pause time analysis.

Dipeptide repeat protein extraction and ELISA

Frozen pellets were lysed in buffer and processed as described previously (Vahsen et al., 2023), and detailed methods are available in the supplemental information.

Confocal imaging and image analysis

Images of markers of cell type and cell stress were obtained at 63x resolution using a Zeiss spinning disk confocal microscope and analyzed using Fiji. Nuclear stains (islet 1, Nkx 6.1) were counted if the nuclear staining was stronger than the cytoplasmic staining. For ChAT and secretagogin, an arbitrary fixed fluorescence intensity threshold was selected for each differentiation, such that no staining was present in non-neuronal cells.

PABP and G3BP were quantified manually by counting the number of cells containing 1 or more bright PABP/G3BP + puncta 0.1–4 μm in diameter (Mahboubi and Stochaj, 2017). The ratio of cytosol/nucleus fluorescence intensity was used as a measure of mislocalization. Images of RNA foci were obtained at 63x using Zeiss LSM 780 and were analyzed in individual slices of the confocal z stack, counting the number of foci per DAPI-stained nucleus.

Viability and toxicity assays

We performed a longitudinal survival and toxicity assay by using the Real-Time Glo MT viability assay (Promega) and the CellTox green cytotoxicity assay (Promega) and performed this at an early time point (DIV 23 in iPSC SNs and DIV 29 in iPSC MNs) and a late time point (DIV 36 in iPSC SNs and DIV 37 in iPSC MNs), taking longitudinal readings every 12–24 h up to 72 h. Data were normalized to the 24-h readout.

RNA sequencing and acquisition of external sequencing data

RNA extraction from iPSC MNs and iPSC SNs was performed using the QIAGEN RNeasy micro kit 5 weeks after final plating from the control cell lines 180, 840, and 856 (both cell types) and the patient lines C9-02-03, C9-04-12 (both cell types), C9-01-06 (MNs), and C9-01-07 (SNs). RNA abundance and quality were assessed using NanoDrop and the Bioanalyzer nano RNA kit. Library preparation was done using paired-end sequencing with a read length of 150 bp. Sequencing was performed on the Illumina NovaSeq 6000. External sequencing datasets were obtained from the Gene Expression Omnibus (GEO) with accession numbers GSE144208, GSE98288 (Hall et al., 2017), GSE201407 (Sommer et al., 2022), GSE203168 (Hawkins et al., 2022), GSE190442 (Yadav et al., 2023), GSE168243 (Nguyen et al., 2021), and GSE76514 (Nichterwitz et al., 2016) or from the dbGaP web site, under phs001158.v2.p1 (Ray et al., 2018). For the single-nucleus spinal cord dataset (GSE190442), the bam data were filtered using the experimental matrix for barcodes assigned to MNs and then converted back to fastq format using samtools.

Sequencing analysis

RNA sequencing analysis was performed using automated pipelines using the cgat-developers software (Cribbs et al., 2019), available at https://github.com/cgat-developers/cgat-flow/ (branch JS-exome). Mapping against Ensembl genome version 101 was performed with STAR using the 2-pass method using a standard hg38 genome, with the following options on first pass: "–alignIntronMin 20 –alignIntronMax 1000000 –alignMatesGapMax 1000000 –alignSJoverhangMin 8 –outFilterMismatchNoverReadLmax 0.04 –outFilterScoreMinOverLread 0.9 –outFilterMatchNminOverLread 0.9". Count tables were obtained using featureCounts for the coding subset of the gene set only (Liao et al., 2014).

Exploratory and differential expression analysis was carried out in R. Differential expression analysis was performed using DESeq2 (Love et al., 2014), with a false discovery threshold of 0.05, and using the model ~group+sex. Log-fold change shrinkage was performed using apeglm (Zhu et al., 2019). GSEA was performed using fgsea (Sergushichev, 2016), and overrepresentation analysis was performed using goseq (Young et al., 2010). Expression correlation was performed using vidger.

Other analyses and illustrations

Analyses for Figures 3, 4, 5, S1, and S3 were produced in GraphPad Prism v9. Comparisons of two groups were performed by two-tailed unpaired t tests and multiple group comparisons by one-way ANOVA with appropriate *post hoc* tests as indicated in the figure legends. Data are presented as single data points and means ± SEM. Differences were considered significant when $p < 0.05$ (*$p < 0.05$; **$p < 0.01$; ***$p < 0.001$; ****$p < 0.0001$; ns: not significant). Illustrations were created with BioRender.com (Figures 2A and 6A) and Microsoft PowerPoint (Figure 1A).

SUPPLEMENTAL INFORMATION

Supplemental information can be found online at https://doi.org/10.1016/j.stemcr.2024.05.008.

ACKNOWLEDGMENTS

J.S. received funding from The Academy of Medical Sciences (SGL025\1095) and the John Fell Oxford University Press Research Fund. A.J.C. was in receipt of the Rosetrees Trust Seedcorn Grant. Y.X. is supported by the Chinese Scholarship Council (CSC) and the Chinese Academy of Medical Sciences (CAMS) Innovation Fund for Medical Science (CIFMS), China (2018-I2M-2-002).

B.F.V. was supported by the University of Oxford Clarendon Fund, St John's College Oxford, the Oxford-MRC Doctoral Training Partnership (MR/N013468/1), and the NIHR Oxford Biomedical Research Centre. R.D. is funded by a Fellowship in Brain Science (University of Oxford); A.M.I. is supported by the UK Dementia Research Institute, through UK DRI Ltd, principally funded by the Medical Research Council, and additional funding partners Alzheimer's Research UK and LifeArc. D.L.B. acknowledges funding from the Wellcome Trust (223149/Z/21/Z) and the Medical Research Council (MR/T020113/1). We thank the Oxford Genomics Centre at the Wellcome Centre for Human Genetics (funded by Wellcome Trust grant reference 203141/Z/16/Z) for the generation and initial processing of the sequencing data. We thank Professor Sally Cowley, Dunn School of Pathology, University of Oxford, for the generation of the cell lines used in this work. This research was funded in part by the Wellcome Trust (203141/Z/16/Z and 223149/Z/21/Z). For the purpose of open access, the authors have applied for a CC BY public copyright license to any author accepted manuscript version arising from this submission.

AUTHOR CONTRIBUTIONS

J.S.: project conceptualization, experimental design and execution, data analysis, bioinformatics, software development, manuscript writing and revision, funding contribution; I.T.-W.: immunofluorescence and western blotting and data analysis (Figures 3, 4, and 5), manuscript contribution; A.J.C.: SN differentiation development/supervision; Y.X.: RNA extraction and sequencing submission; B.F.V.: MN differentiation; M.C.: dipeptide ELISA; R.D.: cell culture advice/development; L.F.: cell culture advice; A.M.I.: dipeptide ELISA development; D.L.B.: SN differentiation development and advice; K.T.: supervision, overall project leadership, funding and laboratory space. All authors read, critically revised, and approved the final manuscript.

DECLARATION OF INTERESTS

The authors declare no competing interests.

Received: August 29, 2023
Revised: May 17, 2024
Accepted: May 17, 2024
Published: June 13, 2024

REFERENCES

Ababneh, N.A., Scaber, J., Flynn, R., Douglas, A., Barbagallo, P., Candalija, A., Turner, M.R., Sims, D., Dafinca, R., Cowley, S.A., and Talbot, K. (2020). Correction of amyotrophic lateral sclerosis related phenotypes in induced pluripotent stem cell-derived motor neurons carrying a hexanucleotide expansion mutation in C9orf72 by CRISPR/Cas9 genome editing using homology-directed repair. Hum. Mol. Genet. *29*, 2200–2217. https://doi.org/10.1093/hmg/ddaa106.

Abo-Rady, M., Kalmbach, N., Pal, A., Schludi, C., Janosch, A., Richter, T., Freitag, P., Bickle, M., Kahlert, A.-K., Petri, S., et al. (2020). Knocking out C9ORF72 Exacerbates Axonal Trafficking Defects Associated with Hexanucleotide Repeat Expansion and Reduces Levels of Heat Shock Proteins. Stem Cell Rep. *14*, 390–405. https://doi.org/10.1016/j.stemcr.2020.01.010.

Andrews, S., Gilley, J., and Coleman, M.P. (2010). Difference Tracker: ImageJ plugins for fully automated analysis of multiple axonal transport parameters. J. Neurosci. Methods *193*, 281–287. https://doi.org/10.1016/j.jneumeth.2010.09.007.

Braems, E., Swinnen, B., and Van Den Bosch, L. (2020). C9orf72 loss-of-function: a trivial, stand-alone or additive mechanism in C9 ALS/FTD? Acta Neuropathol. *140*, 625–643. https://doi.org/10.1007/s00401-020-02214-x.

Clark, A.J., Kugathasan, U., Baskozos, G., Priestman, D.A., Fugger, N., Lone, M.A., Othman, A., Chu, K.H., Blesneac, I., Wilson, E.R., et al. (2021). An iPSC model of hereditary sensory neuropathy-1 reveals L-serine-responsive deficits in neuronal ganglioside composition and axoglial interactions. Cell Reports Medicine *2*, 100345. https://doi.org/10.1016/j.xcrm.2021.100345.

Cribbs, A.P., Luna-Valero, S., George, C., Sudbery, I.M., Berlanga-Taylor, A.J., Sansom, S.N., Smith, T., Ilott, N.E., Johnson, J., Scaber, J., et al. (2019). CGAT-core: a python framework for building scalable, reproducible computational biology workflows. F1000Research *8*, 377. https://doi.org/10.12688/f1000research.18674.1.

Dafinca, R., Barbagallo, P., Farrimond, L., Candalija, A., Scaber, J., Ababneh, N.A., Sathyaprakash, C., Vowles, J., Cowley, S.A., and Talbot, K. (2020). Impairment of Mitochondrial Calcium Buffering Links Mutations in C9ORF72 and TARDBP in iPS-Derived Motor Neurons from Patients with ALS/FTD. Stem Cell Rep. *14*, 892–908. https://doi.org/10.1016/j.stemcr.2020.03.023.

Dafinca, R., Scaber, J., Ababneh, N., Lalic, T., Weir, G., Christian, H., Vowles, J., Douglas, A.G., Fletcher-Jones, A., Browne, C., et al. (2016). C9orf72 Hexanucleotide Expansions Are Associated with Altered Endoplasmic Reticulum Calcium Homeostasis and Stress Granule Formation in Induced Pluripotent Stem Cell-Derived Neurons from Patients with Amyotrophic Lateral Sclerosis and Frontotemporal Dementia. Stem Cell. *34*, 2063–2078. https://doi.org/10.1002/stem.2388.

Feldman, E.L., Goutman, S.A., Petri, S., Mazzini, L., Savelieff, M.G., Shaw, P.J., and Sobue, G. (2022). Amyotrophic lateral sclerosis. Lancet *400*, 1363–1380. https://doi.org/10.1016/S0140-6736(22)01272-7.

Fumagalli, L., Young, F.L., Boeynaems, S., De Decker, M., Mehta, A.R., Swijsen, A., Fazal, R., Guo, W., Moisse, M., Beckers, J., et al. (2021). C9orf72 -derived arginine-containing dipeptide repeats associate with axonal transport machinery and impede microtubule-based motility. Sci. Adv. *7*, eabg3013. https://doi.org/10.1126/sciadv.abg3013.

Gautier, O., Blum, J.A., Maksymetz, J., Chen, D., Schweingruber, C., Mei, I., Hermann, A., Hackos, D.H., Hedlund, E., Ravits, J., and Gitler, A.D. (2023). Human motor neurons are rare and can be transcriptomically divided into known subtypes. Preprint at bioRxiv. https://doi.org/10.1101/2023.04.05.535689.

Geser, F., Brandmeir, N.J., Kwong, L.K., Martinez-Lage, M., Elman, L., McCluskey, L., Xie, S.X., Lee, V.M.Y., and Trojanowski, J.Q. (2008). Evidence of Multisystem Disorder in Whole-Brain Map of Pathological TDP-43 in Amyotrophic Lateral Sclerosis. Arch. Neurol. *65*, 636–641. https://doi.org/10.1001/archneur.65.5.636.

Giacomelli, E., Vahsen, B.F., Calder, E.L., Xu, Y., Scaber, J., Gray, E., Dafinca, R., Talbot, K., and Studer, L. (2022). Human stem cell models of neurodegeneration: From basic science of amyotrophic lateral sclerosis to clinical translation. Cell Stem Cell *29*, 11–35. https://doi.org/10.1016/j.stem.2021.12.008.

Guo, Y.-S., Wu, D.-X., Wu, H.-R., Wu, S.-Y., Yang, C., Li, B., Bu, H., Zhang, Y.-S., and Li, C.-Y. (2009). Sensory involvement in the SOD1-G93A mouse model of amyotrophic lateral sclerosis. Exp. Mol. Med. *41*, 140. https://doi.org/10.3858/emm.2009.41.3.017.

Hall, C.E., Yao, Z., Choi, M., Tyzack, G.E., Serio, A., Luisier, R., Harley, J., Preza, E., Arber, C., Crisp, S.J., et al. (2017). Progressive Motor Neuron Pathology and the Role of Astrocytes in a Human Stem Cell Model of VCP-Related ALS. Cell Rep. *19*, 1739–1749. https://doi.org/10.1016/j.celrep.2017.05.024.

Hawkins, S., Namboori, S.C., Tariq, A., Blaker, C., Flaxman, C., Dey, N.S., Henley, P., Randall, A., Rosa, A., Stanton, L.W., and Bhinge, A. (2022). Upregulation of beta-catenin due to loss of miR-139 contributes to motor neuron death in amyotrophic lateral sclerosis. Stem Cell Rep. *17*, 1650–1665. https://doi.org/10.1016/j.stemcr.2022.05.019.

Heads, T., Pollock, M., Robertson, A., Sutherland, W.H.F., and Allpress, S. (1991). Sensory nerve pathology in amyotrophic lateral sclerosis. Acta Neuropathol. *82*, 316–320. https://doi.org/10.1007/bf00308818.

Ho, R., Workman, M.J., Mathkar, P., Wu, K., Kim, K.J., O'Rourke, J.G., Kellogg, M., Montel, V., Banuelos, M.G., Arogundade, O.A., et al. (2021). Cross-Comparison of Human iPSC Motor Neuron Models of Familial and Sporadic ALS Reveals Early and Convergent Transcriptomic Disease Signatures. Cell Systems *12*, 159–175.e159. https://doi.org/10.1016/j.cels.2020.10.010.

Johnston, C.A., Stanton, B.R., Turner, M.R., Gray, R., Blunt, A.H., Butt, D., Ampong, M.A., Shaw, C.E., Leigh, P.N., and Al-Chalabi, A. (2006). Amyotrophic lateral sclerosis in an urban setting: a population based study of inner city London. J. Neurol. *253*, 1642–1643. https://doi.org/10.1007/s00415-006-0195-y.

Kawamura, Y., Dyck, P.J., Shimono, M., Okazaki, H., Tateishi, J., and Doi, H. (1981). Morphometric comparison of the vulnerability of peripheral motor and sensory neurons in amyotrophic lateral sclerosis. J. Neuropathol. Exp. Neurol. *40*, 667–675. https://doi.org/10.1097/00005072-198111000-00008.

Kihira, T., Yoshida, S., Yoshimasu, F., Wakayama, I., and Yase, Y. (1997). Involvement of Onuf's nucleus in amyotrophic lateral sclerosis. J. Neurol. Sci. *147*, 81–88. https://doi.org/10.1016/S0022-510X(96)05313-0.

Liao, Y., Smyth, G.K., and Shi, W. (2014). featureCounts: an efficient general purpose program for assigning sequence reads to genomic features. Bioinformatics *30*, 923–930. https://doi.org/10.1093/bioinformatics/btt656.

Love, M.I., Huber, W., and Anders, S. (2014). Moderated estimation of fold change and dispersion for RNA-seq data with DESeq2. Genome Biol. *15*, 550. https://doi.org/10.1186/s13059-014-0550-8.

Mahboubi, H., and Stochaj, U. (2017). Cytoplasmic stress granules: Dynamic modulators of cell signaling and disease. Biochim. Biophys. Acta, Mol. Basis Dis. *1863*, 884–895. https://doi.org/10.1016/j.bbadis.2016.12.022.

Mehta, A.R., Gregory, J.M., Dando, O., Carter, R.N., Burr, K., Nanda, J., Story, D., McDade, K., Smith, C., Morton, N.M., et al. (2021). Mitochondrial bioenergetic deficits in C9orf72 amyotrophic lateral sclerosis motor neurons cause dysfunctional axonal homeostasis. Acta Neuropathol. *141*, 257–279. https://doi.org/10.1007/s00401-020-02252-5.

Nguyen, M.Q., von Buchholtz, L.J., Reker, A.N., Ryba, N.J., and Davidson, S. (2021). Single-nucleus transcriptomic analysis of human dorsal root ganglion neurons. Elife *10*, e71752. https://doi.org/10.7554/eLife.71752.

Nichterwitz, S., Chen, G., Aguila Benitez, J., Yilmaz, M., Storvall, H., Cao, M., Sandberg, R., Deng, Q., and Hedlund, E. (2016). Laser capture microscopy coupled with Smart-seq2 for precise spatial transcriptomic profiling. Nat. Commun. *7*, 12139. https://doi.org/10.1038/ncomms12139.

Okamoto, K., Hirai, S., Amari, M., Iizuka, T., Watanabe, M., Murakami, N., and Takatama, M. (1993). Oculomotor nuclear pathology in amyotrophic lateral sclerosis. Acta Neuropathol. *85*, 458–462. https://doi.org/10.1007/bf00230482.

Park, J.W., Vahidi, B., Taylor, A.M., Rhee, S.W., and Jeon, N.L. (2006). Microfluidic culture platform for neuroscience research. Nat. Protoc. *1*, 2128–2136. https://doi.org/10.1038/nprot.2006.316.

Ray, P., Torck, A., Quigley, L., Wangzhou, A., Neiman, M., Rao, C., Lam, T., Kim, J.Y., Kim, T.H., Zhang, M.Q., et al. (2018). Comparative transcriptome profiling of the human and mouse dorsal root ganglia: an RNA-seq-based resource for pain and sensory neuroscience research. Pain *159*, 1325–1345. https://doi.org/10.1097/j.pain.0000000000001217.

Schindelin, J., Arganda-Carreras, I., Frise, E., Kaynig, V., Longair, M., Pietzsch, T., Preibisch, S., Rueden, C., Saalfeld, S., Schmid, B., et al. (2012). Fiji: an open-source platform for biological-image analysis. Nat. Methods *9*, 676–682. https://doi.org/10.1038/nmeth.2019.

Sergushichev, A.A. (2016). An algorithm for fast preranked gene set enrichment analysis using cumulative statistic calculation. Preprint at bioRxiv. https://doi.org/10.1101/060012.

Shi, Y., Lin, S., Staats, K.A., Li, Y., Chang, W.-H., Hung, S.-T., Hendricks, E., Linares, G.R., Wang, Y., Son, E.Y., et al. (2018). Haploinsufficiency leads to neurodegeneration in C9ORF72 ALS/FTD human induced motor neurons. Nat. Med. *24*, 313–325. https://doi.org/10.1038/nm.4490.

Sleigh, J.N., Tosolini, A.P., Gordon, D., Devoy, A., Fratta, P., Fisher, E.M.C., Talbot, K., and Schiavo, G. (2020). Mice Carrying ALS Mutant TDP-43, but Not Mutant FUS, Display In Vivo Defects in Axonal Transport of Signaling Endosomes. Cell Rep. *30*, 3655–3662.e3652. https://doi.org/10.1016/j.celrep.2020.02.078.

Sommer, D., Rajkumar, S., Seidel, M., Aly, A., Ludolph, A., Ho, R., Boeckers, T.M., and Catanese, A. (2022). Aging-Dependent Altered Transcriptional Programs Underlie Activity Impairments in Human C9orf72-Mutant Motor Neurons. Front. Mol. Neurosci. *15*, 894230. https://doi.org/10.3389/fnmol.2022.894230.

Talbot, K. (2014). Amyotrophic lateral sclerosis: cell vulnerability or system vulnerability? J. Anat. *224*, 45–51. https://doi.org/10.1111/joa.12107.

Tao, Q.-Q., Wei, Q., and Wu, Z.-Y. (2018). Sensory nerve disturbance in amyotrophic lateral sclerosis. Life Sci. *203*, 242–245. https://doi.org/10.1016/j.lfs.2018.04.052.

Vahsen, B.F., Gray, E., Thompson, A.G., Ansorge, O., Anthony, D.C., Cowley, S.A., Talbot, K., and Turner, M.R. (2021). Non-neuronal cells in amyotrophic lateral sclerosis — from pathogenesis to biomarkers. Nat. Rev. Neurol. *17*, 333–348. https://doi.org/10.1038/s41582-021-00487-8.

Vahsen, B.F., Nalluru, S., Morgan, G.R., Farrimond, L., Carroll, E., Xu, Y., Cramb, K.M.L., Amein, B., Scaber, J., Katsikoudi, A., et al. (2023). C9orf72-ALS human iPSC microglia are pro-inflammatory and toxic to co-cultured motor neurons via MMP9. Nat. Commun. *14*, 5898. https://doi.org/10.1038/s41467-023-41603-0.

Williamson, T.L., and Cleveland, D.W. (1999). Slowing of axonal transport is a very early event in the toxicity of ALS–linked SOD1 mutants to motor neurons. Nat. Neurosci. *2*, 50–56. https://doi.org/10.1038/4553.

Workman, M.J., Lim, R.G., Wu, J., Frank, A., Ornelas, L., Panther, L., Galvez, E., Perez, D., Meepe, I., Lei, S., et al. (2023). Large-scale differentiation of iPSC-derived motor neurons from ALS and control subjects. Neuron *111*, 1191–1204.e5. https://doi.org/10.1016/j.neuron.2023.01.010.

Yadav, A., Matson, K.J.E., Li, L., Hua, I., Petrescu, J., Kang, K., Alkaslasi, M.R., Lee, D.I., Hasan, S., Galuta, A., et al. (2023). A cellular taxonomy of the adult human spinal cord. Neuron *111*, 328–344.e7. https://doi.org/10.1016/j.neuron.2023.01.007.

Young, M.D., Wakefield, M.J., Smyth, G.K., and Oshlack, A. (2010). Gene ontology analysis for RNA-seq: accounting for selection bias. Genome Biol. *11*, R14. https://doi.org/10.1186/gb-2010-11-2-r14.

Zhang, K., Donnelly, C.J., Haeusler, A.R., Grima, J.C., Machamer, J.B., Steinwald, P., Daley, E.L., Miller, S.J., Cunningham, K.M., Vidensky, S., et al. (2015). The C9orf72 repeat expansion disrupts nucleocytoplasmic transport. Nature *525*, 56–61. https://doi.org/10.1038/nature14973.

Zhang, S., Cooper-Knock, J., Weimer, A.K., Shi, M., Moll, T., Marshall, J.N.G., Harvey, C., Nezhad, H.G., Franklin, J., Souza, C.D.S., et al. (2022). Genome-wide identification of the genetic basis of amyotrophic lateral sclerosis. Neuron *110*, 992–1008.e11. https://doi.org/10.1016/j.neuron.2021.12.019.

Zhu, A., Ibrahim, J.G., and Love, M.I. (2019). Heavy-tailed prior distributions for sequence count data: removing the noise and preserving large differences. Bioinformatics *35*, 2084–2092. https://doi.org/10.1093/bioinformatics/bty895.

Ziff, O.J., Neeves, J., Mitchell, J., Tyzack, G., Martinez-Ruiz, C., Luisier, R., Chakrabarti, A.M., McGranahan, N., Litchfield, K., Boulton, S.J., et al. (2023). Integrated transcriptome landscape of ALS identifies genome instability linked to TDP-43 pathology. Nat. Commun. *14*, 2176. https://doi.org/10.1038/s41467-023-37630-6.

Stem Cell Reports, Volume *19*

Supplemental Information

Cellular and axonal transport phenotypes due to the *C9ORF72* HRE in iPSC motor and sensory neurons

Jakub Scaber, Iona Thomas-Wright, Alex J. Clark, Yinyan Xu, Björn F. Vahsen, Mireia Carcolé, Ruxandra Dafinca, Lucy Farrimond, Adrian M. Isaacs, David L. Bennett, and Kevin Talbot

Supplementary Tables

iPSC line	abbrev.	sex	age	ALS variant	PMID
OX3-06	OX3	male	49	-	29604226
SFC180-03-03	180	female	60	-	28827786
SFC840-03-03	840	female	67	-	26905200
SFC841-03-01	841	male	36	-	27097283
SFC856-03-04	856	female	78	-	28827786
C9-01-07<br>C9-01-06	C9-1	male	72	*C9ORF72*<br>(~970 G_4C_2 repeats)	32330447
C9-02-02<br>C9-02-03	C9-2	female	58	*C9ORF72*<br>(~1000 G_4C_2 repeats)	27097283
C9-04-12<br>C9-04-01	C9-3	male	39	*C9ORF72*<br>(>500 G_4C_2 repeats)	32330447

Supplementary Table 1: iPSC lines used in this study. Clones from the same patient are grouped. Abbreviations: PMID = PubMed identification number; ALS = amyotrophic lateral sclerosis

Antibody	Species	Dilution	Source	Catalogue number
β-Actin	Ms	1:10000 WB	Invitrogen	AM4302
β-Actin	Rb	1:10000 WB	Abcam	Ab227387
Advillin/p92	Rb	1:1000 WB	Abcam	Ab72210
Bax	Rb	1:1000 WB	Abcam	Ab32503
Brn3a	Rb	1:500 WB	Merck	MAB5945
Brn3a	Ms	1:100 WB	Merck	MAB1585
C9orf72	Ms	1:500 WB	GeneTex	GTX632041
Caspase 3	Rb	1:1000 WB	Cell Signalling Technology	9662S
ChAT	Gt	1:1000 WB<br>1:100 IF	Merck	AB144P
ChAT	Rb	1:1000 WB<br>1:1000 IF	Abcam	ab178850
Cleaved Caspase-3	Rb	1:1000 IF<br>1:1000 WB	Cell Signalling Technology	9661S
G3BP	Ms	1:1000 IF	Abcam	ab56574
Hb9	Ms	1:1000 WB	DSHB	81.5C10-s
Islet-1	Ms	1:1000 WB	DSHB	40.206
Nkx6.1	Ms	1:300 WB<br>1:200 IF	DSHB	F55A12
PABP	Rb	1:500 IF	Abcam	ab21060
PUMA	Rb	1:1000 WB	Proteintech	55120-1-AP
TDP-43	Rb	1:200 IF	ProteinTech	10782-2-AP
Tuj1	Rb	1:1000 IF	Abcam	ab15568
Tuj1	Ch	1:1000 IF	Abcam	ab107216

Supplementary Table 2: Primary antibodies used for immunofluorescence and western blotting. Abbreviations: Ch = chicken; Gt = goat; IF = immunocytochemistry; Ms = Mouse; Rb = Rabbit; WB = western blotting.

Antibody	Species	Dilution	Source	Catalogue number
Anti-rabbit AlexaFluor647	Dy	1:1000 IF	Invitrogen	A31573
Anti-rabbit AlexaFluor546	Dy	1:1000 IF	Invitrogen	A10040
Anti-rabbit AlexaFluor488	Dy	1:1000 IF	Invitrogen	A21206
Anti-chicken AlexaFluor647	Gt	1:1000 IF	Invitrogen	A21449
Anti-mouse AlexaFluor568	Gt	1:1000 IF	Invitrogen	A10037
Anti-mouse AlexaFluor488	Gt	1:0000 IF	Invitrogen	A11001
Anti-guineapig AlexaFluor568	Gt	1:1000 IF	Invitrogen	A11075
Anti-goat AlexaFluor647	Dy	1:1000 IF	Invitrogen	A21447
Anti-mouse IgG	Dy	1:5000 WB	LI-COR	926-32212
Anti-mouse IgG HRP	Sh	1:5000 WB	Amersham	NA934V
Anti-rabbit IgG	Dy	1:5000 WB	LI-COR	926-68073
Anti-rabbit IgG HRP	Dy	1:5000 WB	Amersham	NA931V
Anti-goat IgG HRP	Dy	1:5000 WB	Invitrogen	PA1-28664

Supplementary Table 3: Secondary antibodies used for immunofluorescence and western blotting. Abbreviations: Dy = donkey; Gt = goat; HRP = horseradish peroxidase; IF = immunocytochemistry; Sh = Sheep; WB = western blotting.

Growth Factor	Source	Catalogue number
Glial-Derived Neurotrophic Factor (GDNF)	Preprotech	450-10
Brain Derived Neurotrophic Factor (BDNF)	Preprotech	450-02
beta-Nerve Growth Factor (NGF)	Preprotech	450-01
Neurotrophin-3 (NT-3)	Preprotech	450-03

Supplementary Table 4: Growth factors used during differentiation.

Supplementary Methods

Motor neuron differentiation

Cryopreserved iPSCs were plated onto Geltrex and grown in mTeSR1 (StemCell Technologies) until 80% confluency, at which timepoint the differentiation was induced (DIV 0). Throughout the differentiation cells were maintained in basal media containing Dulbecco's Modified Eagle's Medium/nutrient mixture F-12 (DMEM/F-12) and Neurobasal medium in a 1:1 ratio, supplemented with 2% B27, 1% N2, ascorbic acid (0.5µM), Anti-Anti and β-mercaptoethanol (50nM) (all ThermoFisher). On DIV 0-3, wnt pathway agonist Chir99021 (3µM; Tocris Bioscience) and AMPK antagonist Compound C (1µM; Merck) were present to induce spinal MN progenitors. Retinoic acid (RA, 1µM; Sigma) and smoothened agonist (SAG, 500nM; Tocris Bioscience) were added on DIV 2. Between DIV 0 and 9 media changes (100%) were performed daily. On DIV 9, cells were passaged 1:3 using Accutase (ThermoFisher), and then fed 3 times per week with basal medium supplemented with RA and SAG.

On DIV 18, cells were dissociated with accutase, counted using trypan blue, and replated onto 0.07% polyethyleneimine (PEI; Merck)/Geltrex. A density of ~1,000,000 cells per well was used for 6-well plates and ~100,000 cells per well for 24-well plates. Basal media was supplemented with rock inhibitor, and γ-secretase inhibitor DAPT (10µM; Tocris Bioscience) was added to promote maturation. Media also contained growth factors BDNF and GDNF (both 10ng/ml; PreproTech). On DIV 19 cytosine arabinoside (AraC, 1µM; Sigma) was added. On DIV 23 AraC and DAPT were removed and mouse laminin (0.5µg/ml; ThermoFisher) was added for subsequent three times weekly 50% media changes. Motor neuron pellets and coverslips were collected five weeks after final plating (DIV 53). A schematic of the differentiation can be found in Figure 1A.

Sensory Neuron Differentiation

Cryopreserved iPSCs were thawed and grown in mTeSR until ~80% confluent. The sensory neuron differentiation followed a previously published protocol[23], by switching to KSR media (KnockOut™ DMEM/F-12, 15% KnockOut Serum Replacement; 1% GlutaMAX, 1% non-essential amino acids, 100µM β-mercaptoethanol and 1% Anti-Anti) containing SMAD inhibitors SB431542 (10µM; Sigma) and LDN-193189 (100nM; Sigma). On DIV 2 Chir99021 (3µM), DAPT (10µM) and FGF signalling inhibitor SU5402 (10µM; Sigma) was added. On DIV 6 dual SMAD inhibition was withdrawn. Between DIV 4 and 10 basal media was switched in 25% increments from KSR media to N2 Media (Neurobasal media, 2% B27 supplement, 1% N2 supplement, 1% GlutaMAX and 1% Anti-Anti). From DIV 0-11 media changes (100%) were performed daily.

On DIV 11 cells were dissociated with TrypLE (ThermoFisher), counted and replated onto PEI/Geltrex coated plates as described for MNs. SU and DAPT were removed and media was supplemented with growth factors NGF (25ng/ml; Peprotech), BDNF (25ng/ml), GDNF (25ng/ml) and Neurotrophin-3 (25ng/ml; Peprotech). As for MNs, rock inhibitor was added on the DIV of splitting and AraC was present between DIV 11 and 13. On DIV 13 AraC was

removed and laminin was added to the media. From DIV 13 to media changes (50%) were performed three times a week. From around DIV 30 the concentration of all four growth factors was reduced to 10ng/ml. Sensory neuron pellets and coverslips were collected five weeks after final plating (DIV 45). A schematic of the differentiation can be found in Figure 1A.

Protein extraction and Quantification

After a wash with PBS, cells were collected in a small volume of phosphate buffer saline (PBS) and pelleted at 3000rpm for 5 minutes at 4°C. Cells were lysed in RIPA buffer (Sigma) and protease inhibitors (Roche) following mechanically homogenisation. After 30 minutes lysates were centrifuged at 10,000rpm for 5 minutes at 4°C and the pellets were discarded. Total protein concentration of the supernatant was determined using the bicinchoninic acid assay (Sigma) according to the manufacturer's instructions.

Western blotting

For gel electrophoresis, protein in RIPA buffer was mixed with NuPAGE LDS sample buffer and NuPAGE sample reducing agent and heated to 95°C for 5 minutes, except where indicated. 10µg per well was loaded on NuPAGE 4-12% Bis-Tris gels (ThermoFisher) and then run at 100V for 120 minutes. Resolved protein was transferred to nitrocellulose membranes using the iBlot 2 Gel Transfer Device (ThermoFisher). Blots were blocked for 1 hour at room temperature in Tris-buffered saline (TBS) containing 0.1% Tween-20 and 5% skimmed milk and incubated overnight at 4°C with primary antibodies (Table 2) in TBS + 0.1% Tween-20 + 1% skimmed milk. Blots were washed three times and incubated with secondary antibody (Supplementary Table 3) in TBS + 0.1% Tween-20 + 1% skimmed milk for 1 hour at room temperature. Blots were washed a further three times before visualisation on a Bio-Rad ChemiDoc. Integrated optical density of the protein bands was measured using Fiji and normalised to β-actin for the same sample.

Immunocytochemistry

Cells grown on glass coverslips were fixed in 4% methanol-free paraformaldehyde for 30 minutes and washed three times with PBS. Coverslips were blocked and permeabilised (0.1% Triton-X; Dow, and 5% donkey serum; BioRad, in PBS) for 1 hour and then incubated overnight with primary antibody in antibody buffer (0.1% Triton-X and 1% donkey serum in PBS). Samples were rinsed in wash buffer (0.1% Triton-X in PBS) for ten minutes and then incubated with secondary antibodies in antibody buffer for one hour. Coverslips were washed again and then incubated with 4',6-Diamidino-2-Phenylindole (DAPI, 1:10,000 dilution in PBS) for ten minutes, followed by a PBS-only wash. Coverslips were mounted in Prolong Diamond Antifade mountant (ThermoFisher).

DPR extraction and ELISA

Frozen cell pellets were lysed using RIPA buffer containing an EDTA-free protease inhibitor cocktail (2x, Sigma) and 10% sodium dodecyl sulphate (SDS, ThermoFisher) and sonicated at 30% AMP for 3 x 10 seconds with 5 s-intervals. The probe was cleaned with ethanol between samples. Samples were centrifuged at 17,000xg for 20 minutes at 16°C and the pellets were discarded. Total protein

concentration of the supernatant was determined using a BCA assay and all samples were adjusted to 0.5mg/ml. Plates were coated with unlabelled anti-poly (GP) or anti-poly (GA) antibodies. After blocking, samples were loaded at 45 µg of protein per well for poly (GP) and 27 µg for poly (GA). Biotinylated anti-poly (GP) and anti-poly (GA) antibodies were used as detectors, followed by sulfo-tagged streptavidin (Meso Scale Discovery, R32AD). Plates were read with the MSD reading buffer (Meso Scale Discovery, R92TC) using the MSD Sector Imager 2400. Signals correspond to intensity of emitted light upon electrochemical stimulation of the assay plate. Prior to analysis, the average reading from a calibrator containing no peptide was subtracted from each reading.

Fluorescence in situ hybridisation

MNs and SNs were fixed in 4% paraformaldehyde for 30 minutes and washed three times with PBS. All work was carried out in an RNase free environment, using RNase ZAP (ThermoFisher). Cells were permeabilised with 0.1% Triton-X and then washed three times in PBS. Tissue was dehydrated with serial addition of 70%, 90% and 100% ethanol and then allowed to air dry before rehydration in PBS followed by 2x Standard Sodium Citrate (SSC) buffer (Sigma). Coverslips were incubated at 80°C for 45 minutes with pre-hybridisation solution (50% Formamide; ThermoFisher and 2x SSC in distilled water) followed by two hours in hybridisation solution (2x SSC, 0.016% BSA; ThermoFisher, 0.8mg/ml salmon sperm DNA; ThermoFisher, 0.8 mg/ml yeast tRNA; ThermoFisher, 8% Dextran sulphate; Amresco, 50% Formamide, 1.6 mM Vanadyl Ribonucleotide; BioLabs, 5mM EDTA and 0.2ng/µl Cy-3-conjugated 2'O-methyl sense oligonucleotide RNA probe; Integrated DNA Technologies) and coverslips were incubated at 80°C for 2 hours. Coverslips were washed three times for 30 minutes each in washing buffer (50% formamide, 0.5x SSC in distilled water). The hybridisation solution was then made up again but using the AlexaFluor488-conjugated antisense oligonucleotide probe (0.2ng/µl) and coverslips were incubated at 80°C for a further 2 hours, followed by three washes in washing buffer as before. Coverslips were washed for 10 minutes three times in 0.5x SSC and finally washed once with PBS. Samples were blocked for 30 minutes (10% donkey serum, 0.1% Triton-X in PBS) and stained with DAPI and Tuj1 according to the immunofluorescence protocol described above.

Manufacture of microfluidic devices

PDMS prepolymer and catalyst (10:1) were mixed thoroughly and poured into a master mould, and subsequently cured in an oven for 2h at 40 °C. Cured PDMS was removed from the master mould and 8 mm reservoirs were punched out. Irreversible bonding was achieved using a plasma cleaner.

Supplementary Figures

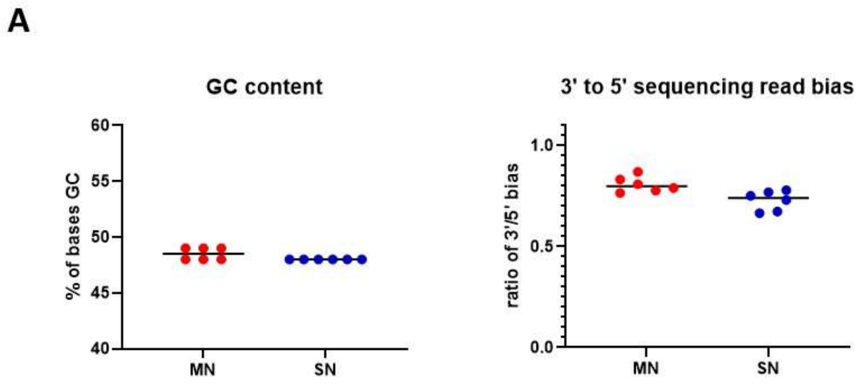

Supplementary Figure 1: Quality control of sequencing data generated in this study. GC content and 3'/5' ratio of sequencing coverage shows small differences only.

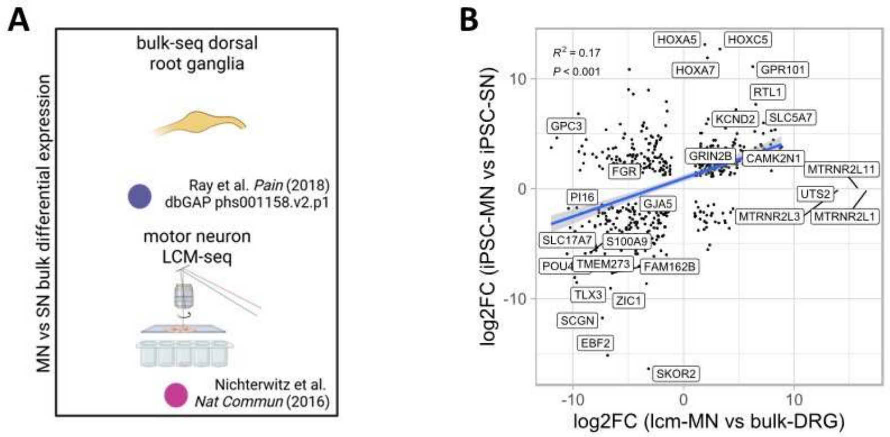

Supplementary Figure 2: Comparison of iPSC-MN vs SN differential expression with bulk datasets confirms findings from single cell data. A: Experimental design showing sources for bulk motor and sensory sequencing datasets used to determine differential expression between these two cell types. **B:** Scatter plot and linear correlation of differentially expressed genes between motor and sensory iPSC neurons versus differential expression between the bulk adult datasets, demonstrate a significant correlation between the two comparisons.

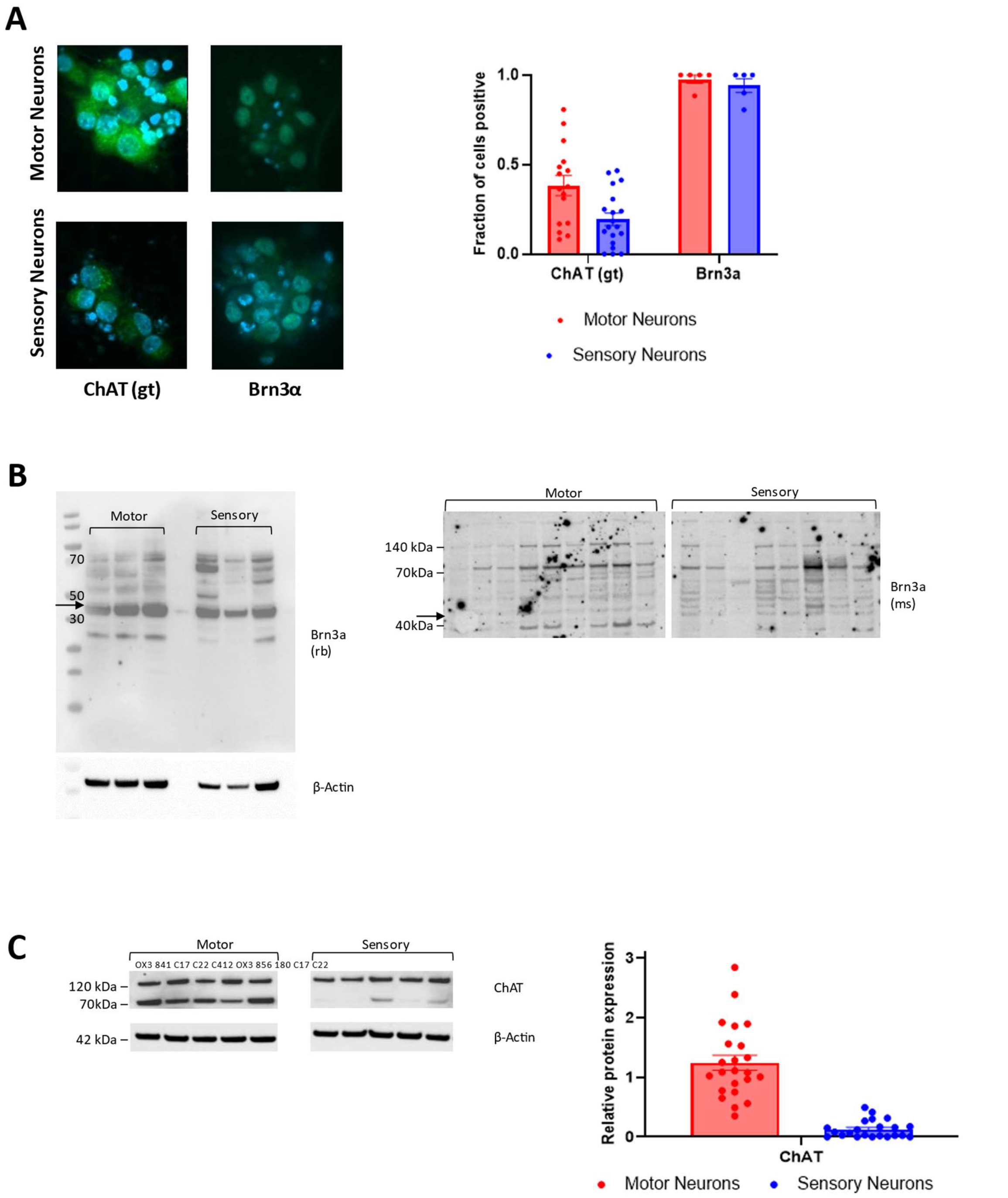

Supplementary Figure 3: Additional immunofluorescence and western blot analysis of motor and sensory neuron markers. A: Immunofluorescence analysis of Brn3a (rabbit) antibody showing ubiquitous staining in both iPS-MNs and iPS-SNs (n=1 differentiation), as well as additional ChAT staining (goat) staining with ChAT+ cells counted according to a fluorescence threshold confirming the presence of a higher proportion of ChAT+ cells in iPSC-MN cultures compared with iPSC-SN cultures ($p < 0.05$, t-test, n=2 differentiations). **B:** Example western blots for Brn3a rabbit (rb) and mouse (ms) antibodies showing a nonspecific staining pattern. **C:** Example western blot and quantification of ChAT staining

with ChAT (goat), which shows a specific band at the expected molecular weight and a nonspecific band at 120kDa. Quantification of the 70kDa band confirms enrichment of ChAT in motor neurons using this antibody ($p < 0.05$, t-test).

A

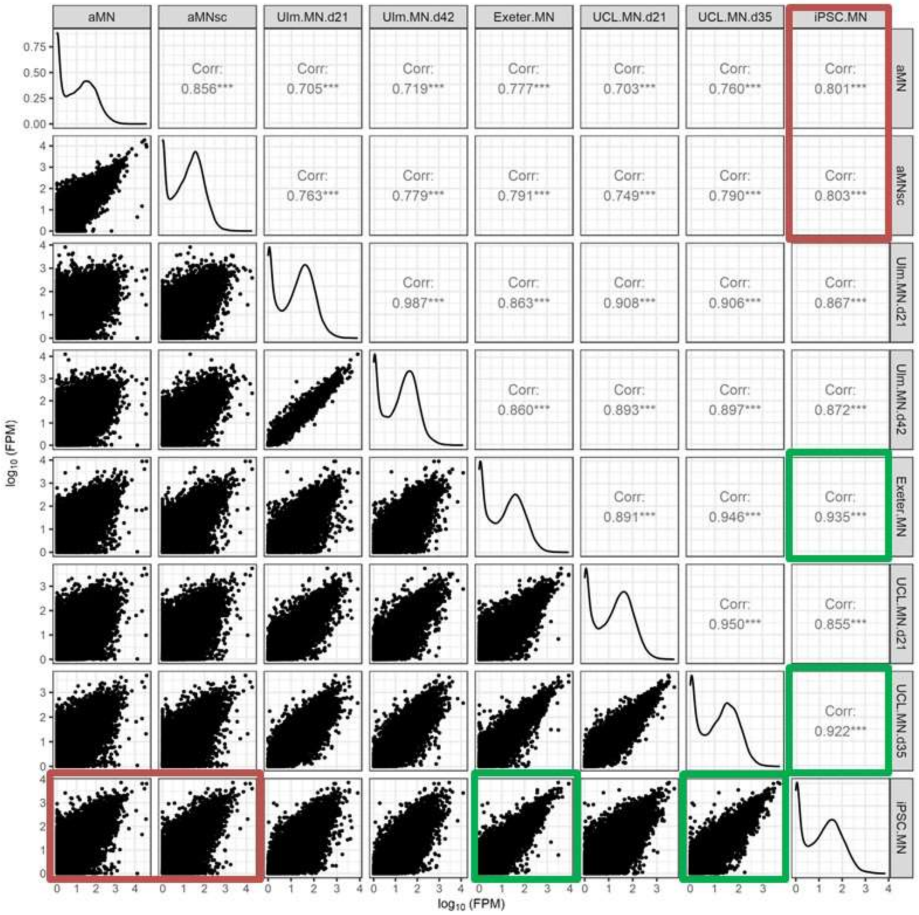

B

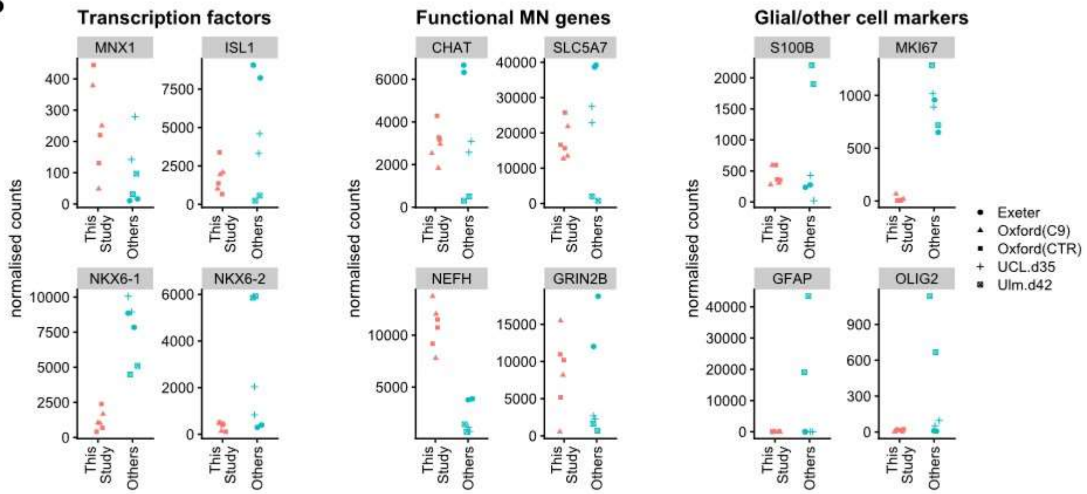

Supplementary Figure 4: Comparison of motor neuron differentiation in this study with previously published iPSC-MN datasets confirms similarity with other differentiations. A: High correlation between this study (labelled iPSC.MN) and two other mature motor neuron cultures (see green squares). Compared to other differentiations, the correlation with adult motor neurons derived from LCM-seq (aMN) and spinal cord single nucleus-seq (aMNsc) compared well with the other datasets (see red squares). Only control datasets were used

for this figure. **B.** Plot of normalised count from DESeq2 object shows similar levels of motor neuron transcription factors as well as expression of functional genes including ChAT, its transporter, neurofilament and ionotropic glutamate receptors. Our differentiation had low levels of non-neuronal cells and comparatively low levels of the proliferation marker *MKI67*. Exeter.MN = GSE203168, UCL.MN = GSE98288, Ulm.MN = GSE201407, aMN= GSE76514, aMNsc = GSE190442.

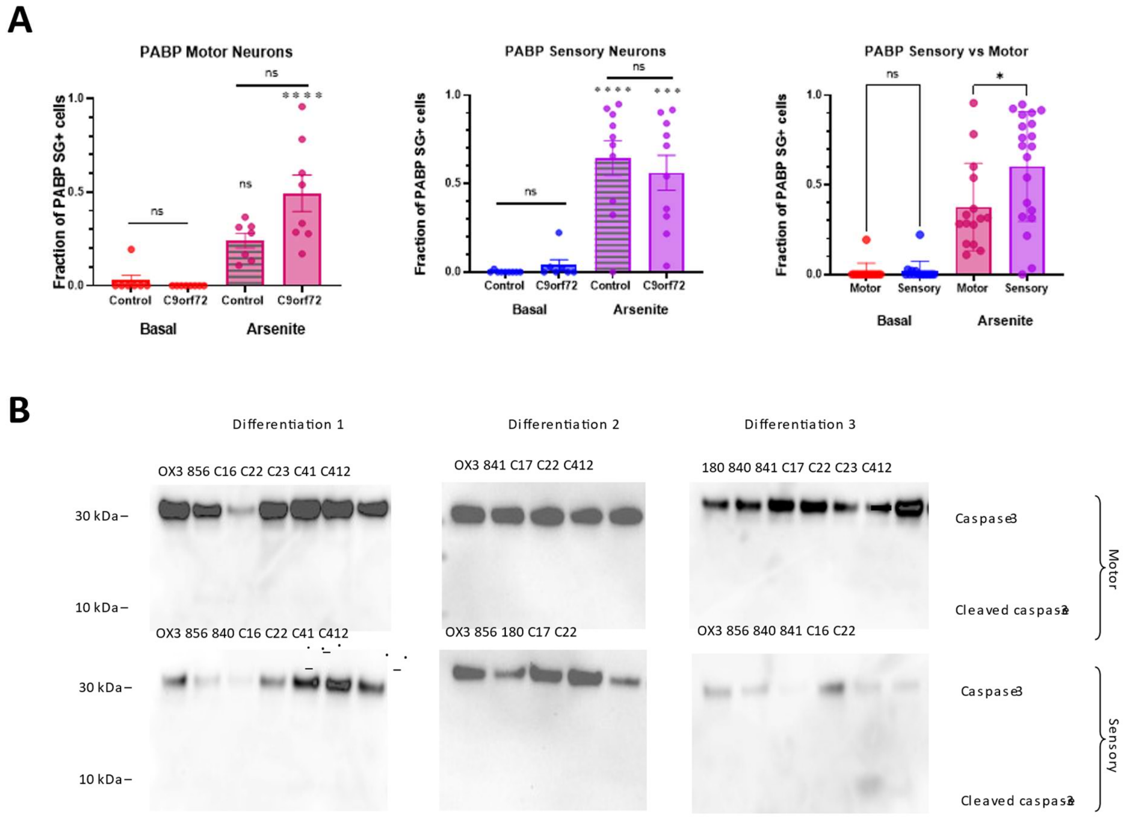

Supplementary Figure 5: Additional cellular phenotypes due to the *C9ORF72* HRE expansion in iPSC-MNs and iPSC-SNs. A: No difference in arsenite-induced PABP+ stress granule (SG) assembly between controls and *C9ORF72*+ iPSC-MNs or iPSC-SNs. Independent of the mutation, iPSC-SNs had a higher frequency of SGs following 1h Arsenite stress than iPSC-MNs. Stars denote significant differences between the basal stressed condition, n=2 differentiations. **B:** No staining of for cleaved caspase-3 (10kDa) when staining for full length caspase 3. n=3 differentiations.

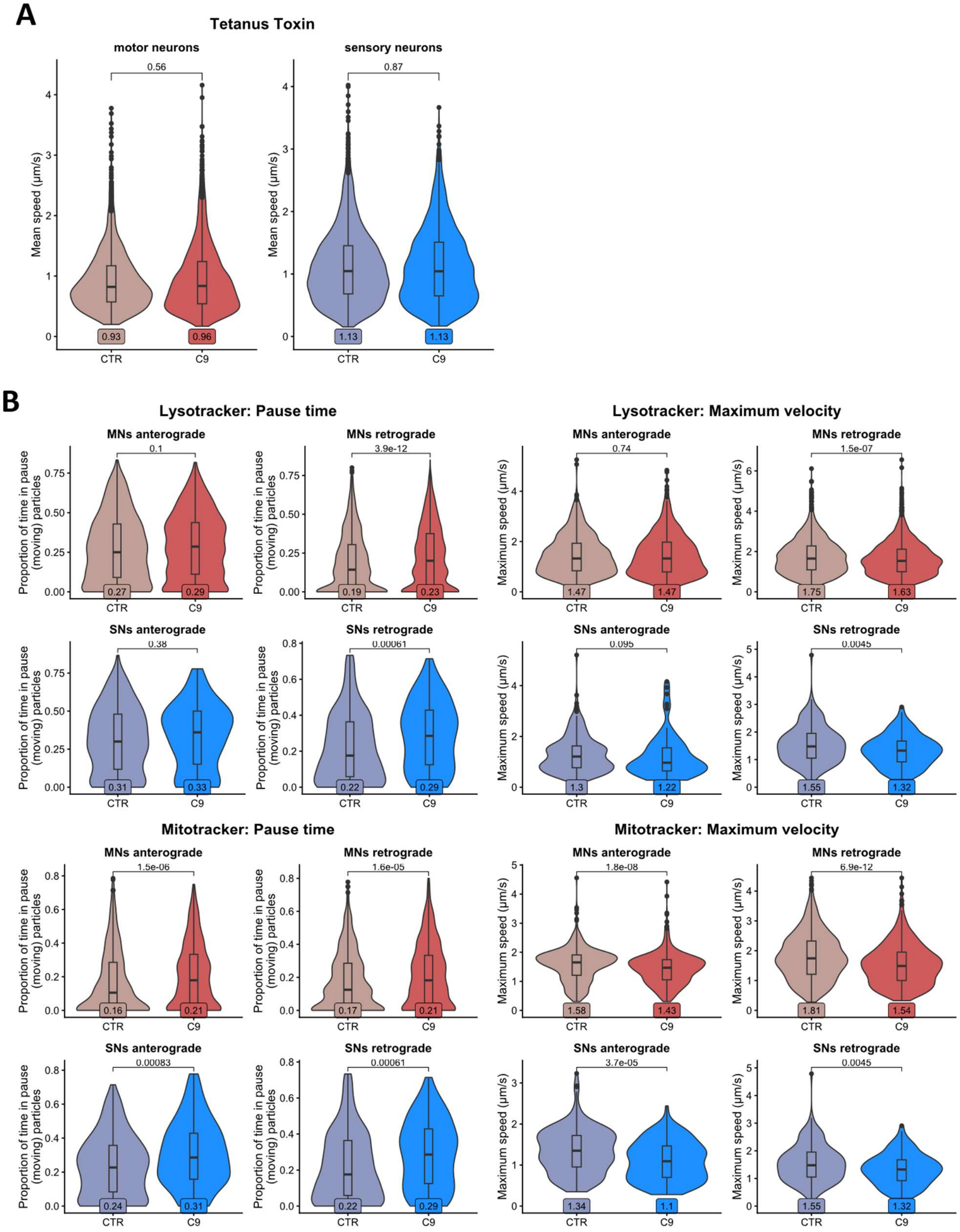

Supplementary Figure 6: Axonal transport of tetanus toxin and additional Lysotracker and Mitotracker data. A: No difference between controls and *C9ORF72*+ iPSC-MNs or iPSC-SNs in retrograde axonal transport of fluorescently labelled tetanus toxin. **B:** Increase in pause

times and decrease maximum velocity occur in lysotracker retrograde transport and Mitotracker bidirectional transport.
